# Higher-dimensional embedding of time-series data for machine learning

**DOI:** 10.3389/frai.2026.1874928

**Published:** 2026-09-25

**Authors:** Karan Singh, Pingal Pratyush Nath, Urbasi Sinha, Aninda Sinha

**Affiliations:** 1 Centre for High Energy Physics, Indian Institute of Science, Bengaluru, Karnataka, India; 2 Raman Research Institute, Bengaluru, Karnataka, India; 3 Department of Physics and Astronomy, University of Calgary, Calgary, AB, Canada

**Keywords:** CNN, ECG, image encoding, orthogonal polynomials, time-series embedding

## Abstract

**Introduction:**

Deep learning has revolutionized image analysis, yet most clinical biosignals, especially multi-lead electrocardiograms (ECGs), remain one-dimensional and awkward for modern vision models.

**Methods:**

We introduce an orthogonal-polynomial imaging (OPI) framework that encodes 12-lead ECGs into a single two-dimensional image through an invertible transformation prior to coarse-graining, producing compact image representations that retain sufficient information for accurate classification. We benchmark the proposed representation against established architectures including ResNet, Transformers, and 1D temporal convolutional models on large-scale PhysioNet ECG datasets.

**Results:**

Our results demonstrate that OPI-based image representations achieve highly competitive classification performance while preserving diagnostically relevant temporal and cross-lead information.

**Discussion:**

The framework bridges raw multi-channel biosignals and standard image-based learning architectures without representation loss prior to coarse-graining, offering an effective, scalable, and physics-inspired representation for automated cardiac diagnostics.

## 1 Introduction

Machine learning (Sarker, 2021; Zhou, 2021; Alpaydin, 2021; Jordan and Mitchell, 2015; El Naqa and Murphy, 2015; Pandey et al., 2019; Bi et al., 2019) has become a cornerstone of modern medical diagnostics, delivering significant improvements in accuracy and efficiency and thereby enabling early detection across diverse healthcare applications (Kononenko, 2001; Oza et al., 2021; Rajula et al., 2020; Bhavsar et al., 2021a,b; Shehab et al., 2022; Picardi et al., 2019; Giger, 2018; Erickson et al., 2017; Sidey-Gibbons and Sidey-Gibbons, 2019; Pumplun et al., 2021; Smiti, 2020; Fatima and Pasha, 2017). In the field of medical imaging, machine learning algorithms, particularly deep learning, have demonstrated exceptional effectiveness, often matching or surpassing human expertise in tasks such as tumor detection and disease classification (Erickson et al., 2017; Giger, 2018; Weimann and Conrad, 2021; Barragán-Montero et al., 2021; Varoquaux and Cheplygina, 2022; Currie et al., 2019; Oakden-Rayner et al., 2020; Willemink et al., 2020; Latif et al., 2019; Kaissis et al., 2020; Alwaeli et al., 2025; Abbood et al., 2022, 2021). This capability to rapidly and accurately analyze complex visual data has made machine learning an indispensable tool for improving patient care and outcomes.

Despite these successes, many physiological signals—including electrocardiograms (ECGs)—are recorded as one-dimensional time series, where conventional analysis relies on handcrafted features or independent lead-by-lead processing. Such approaches often fail to fully capture the intricate spatiotemporal dependencies among signals. While deep neural networks can operate directly on raw time series, image-based representations provide an alternative means of exploiting spatial inductive biases and can achieve competitive performance on certain tasks. This discrepancy motivates a shift toward leveraging image processing techniques for problems traditionally rooted in one-dimensional signal analysis. The central challenge, however, is to transform one-dimensional signals into structured representations suitable for image processing, while preserving the inherent information and physiological structure. We will refer to this representation as Orthogonal Polynomial Imaging–OPI for short.

To address this, we introduce a novel encoding framework inspired by the partial wave decomposition of scattering amplitudes routinely used in high energy physics. Our method maps multiple one-dimensional signals into a unified two-dimensional representation. By superposing different signals using orthogonal polynomials, we generate compact images that embed both temporal dynamics and inter-lead relationships. This representation enables the direct use of advanced image classification models and facilitates the integration of diverse data sources into a single coherent structure (Figure 1).

**Figure 1:**
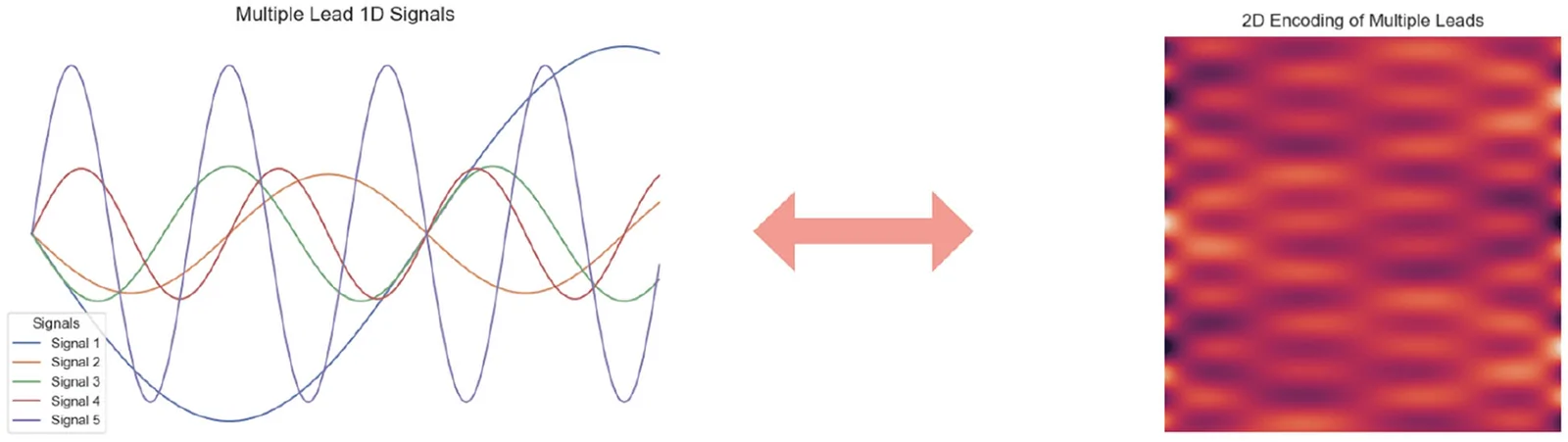
Encoding of multiple one dimensional leads to two dimensional images using Legendre Polynomials.

The relevance of this approach is particularly evident for the 12-lead ECG (Aziz et al., 2021; Kachuee et al., 2018; Weimann and Conrad, 2021; Śmigiel et al., 2021; Jambukia et al., 2015; Alfaras et al., 2019; Pyakillya et al., 2017; Houssein et al., 2017; Kiranyaz et al., 2015b,a; Mathews et al., 2018; Liu X. et al., 2021; Ribeiro et al., 2020; Yoon and Joo, 2024), the clinical gold standard for non-invasive cardiac assessment. Each lead provides a distinct projection of the heart's electrical activity, and accurate diagnosis requires synthesizing information across all 12. Analyzing these signals independently may overlook subtle inter dependencies, whereas our method integrates them naturally into a single image. This not only improves diagnostic accuracy but also provides a holistic view of cardiac electrophysiology. In this paper, we present a comprehensive evaluation of our encoding framework by classifying 12-lead ECG data from PhysioNet database and benchmarking our strategy against existing methods. Although our focus is on ECGs, the approach is general and can be extended to other multi-channel time-series data with similar characteristics.

### 1.1 12-Lead ECG

For over a century, the 12-lead ECG (Khunti, 2014; Garcia and Holtz, 2001; Corrado et al., 2009) has been the primary tool for assessing cardiac electrical function, providing a rapid, inexpensive, and widely accessible means to diagnose various cardiovascular conditions. Using ten electrodes on the limbs and chest, it records 12 voltage signals which are distinct projections of the heart's electrical field offering a detailed spatiotemporal view of cardiac activity.

These 12 leads are anatomically organized to capture electrical vectors in two principal planes: the frontal plane, represented by the limb leads (I, II, III, aVR, aVL, aVF), and the horizontal plane, captured by the six precordial chest leads (V1–V6). This dual-plane perspective allows clinicians to localize myocardial ischemia, detect conduction blocks, and interpret complex arrhythmias with spatial specificity. Each lead contributes a unique window into the electrophysiological state of specific cardiac regions, making the 12-lead ECG an indispensable tool for diagnosing conditions ranging from atrial fibrillation and ventricular tachycardia to myocardial infarction and inherited channelopathies.

At the heart of ECG interpretation is the P-QRS-T waveform, which encodes the sequential electrical events of the cardiac cycle. The P wave reflects atrial depolarization, the QRS complex corresponds to the rapid depolarization of the ventricles, and the T wave represents ventricular repolarization. Clinical interpretation hinges not only on the morphology and amplitude of these components but also on the timing between them. Intervals such as the PR segment (atrial-to-ventricular conduction), ST segment (isoelectric period following depolarization), and QT interval (duration of ventricular electrical activity) carry critical diagnostic significance. For example, a left bundle branch block is identified by a QRS duration exceeding 120 milliseconds, along with specific waveform changes in lateral leads such as I, V5, and V6.

Despite its diagnostic richness, interpreting a 12-lead ECG is inherently complex (Drezner et al., 2012; Davies et al., 2019; Hill et al., 2011; Cook et al., 2020; Schläpfer and Wellens, 2017; Kundu et al., 2000; Sia et al., 2022; Kashou et al., 2023; Alday et al., 2020; Tahri Sqalli et al., 2021; Hasitha et al., 2025; Garcia and Holtz, 2001; Khunti, 2014). Clinicians must synthesize information across multiple leads, correlate electrical signatures with anatomical context, and distinguish between normal variants and pathological findings, all while accounting for confounders like baseline noise, artifacts, or comorbidities. This level of interpretation requires years of training and is subject to variability between readers, often leading to missed or delayed diagnoses in clinical practice.

In this setting, automation through artificial intelligence becomes not only advantageous but essential. The 12-lead ECG is uniquely suited for machine learning applications due to its structured yet information-dense nature, combining temporal dynamics with spatially distributed features. AI models can be trained to recognize subtle patterns across leads, detect rare syndromes, and triage critical abnormalities with speed and consistency. By embedding expert-level interpretation into scalable software systems, AI has the potential to democratize access to cardiac diagnostics, reduce human error, and support timely clinical decision-making—especially in high-volume or resource-limited settings.

### 1.2 Deep learning architectures for automated ECG interpretation

The 12-lead ECG's rich spatiotemporal structure and multidimensional signal make it particularly well-suited for deep learning. Traditionally, ECG interpretation relied on handcrafted features derived from domain knowledge, such as RR intervals, QRS durations, and ST-segment deviations, fed into classical machine learning pipelines. However, in recent years, this paradigm has shifted dramatically with the advent of deep learning models that operate directly on raw or minimally preprocessed waveforms. These end-to-end approaches learn task-specific representations automatically, often capturing subtle or subclinical patterns that may be imperceptible to human experts (Hannun et al., 2019; Rajpurkar et al., 2022; Silipo and Marchesi, 2002). This shift has been propelled by several key innovations. Contrastive learning techniques (Soltanieh et al., 2022; Wei et al., 2022; Le et al., 2023; Diamant et al., 2022; Ammour et al., 2023; Wang N. et al., 2023; Chen et al., 2021; Rabbani and Khan, 2022; Sangha et al., 2024; Li et al., 2022) have enabled models to learn discriminative features from unlabeled ECG data by contrasting similar and dissimilar samples. Self-supervised learning and pretraining strategies (Liu H. et al., 2021; Weimann and Conrad, 2025; Liu et al., 2025; Mehari and Strodthoff, 2022; Ma et al., 2025; Oh et al., 2022; Zhu et al., 2025; Gedon et al., 2021; Hu et al., 2023) have further improved generalization by leveraging large-scale unannotated datasets. In parallel, attention-based architectures (Kuvaev and Khudorozhkov, 2019; Huang et al., 2024; Zhang et al., 2020; Wang et al., 2021; Singh and Sharma, 2022; Sobahi et al., 2022; Yao et al., 2020; Lu et al., 2023; Islam et al., 2023; Wang Y. et al., 2023) have enhanced model interpretability and performance by dynamically focusing on the most relevant temporal or spatial regions of the signal.

#### 1.2.1 Convolutional neural networks : capturing morphology

Convolutional Neural Networks (CNNs) form the backbone of ECG classification models due to their ability to hierarchically learn key morphological features such as wave amplitude, duration, and slope (LeCun et al., 2015; Baloglu et al., 2019; Ozaltin and Yeniay, 2023; Ullah et al., 2021). Approaches range from simple 1D-CNN architectures for ECG classification (Ahmed et al., 2023; Chourasia et al., 2020) to more advanced models that integrate *Recurrent Neural Networks* for improved temporal modeling and classification accuracy (Cheng et al., 2021; Xu et al., 2020). Generic CNNs have also been successfully adapted for ECG analysis on wearable devices (Li et al., 2018), and some have been further enhanced using wavelet transforms to better capture time-frequency information (Zhao et al., 2020). Given the black-box nature of deep learning models, recent efforts such as the CNN Explanations Framework for ECG Signals (CEFEs) have focused on providing interpretable insights into how CNN-based models make decisions (Maweu et al., 2021).

#### 1.2.2 Recurrent neural networks

Recurrent Neural Networks (RNNs), particularly Long Short-Term Memory (LSTM) and Gated Recurrent Unit (GRU) variants, are well-suited for modeling temporal dependencies in ECG signals, both across and within cardiac cycles (Wang et al., 2019). Their strength lies in capturing sequential patterns, making them especially effective for tasks like arrhythmia classification and atrial fibrillation detection, where inter-beat relationships play a crucial role (Singh et al., 2018; Übeyli, 2009; Zhang et al., 2017).

To leverage both spatial and temporal information, hybrid architectures that combine Convolutional Neural Networks (CNNs) with RNNs have gained popularity. These models first extract morphological features using CNN layers and then model temporal dynamics through RNNs, enabling more comprehensive ECG analysis (Guo et al., 2019; Übeyli, 2010; Xiong et al., 2018; Lui and Chow, 2018; Zihlmann et al., 2017; Pokaprakarn et al., 2021; Limam and Precioso, 2017; Zhang et al., 2020).

Beyond classification, RNNs have also been explored in a range of ECG-related applications, including biometric identification (Salloum and Kuo, 2017; Kim and Pyun, 2020), and sleep apnea detection (Cheng et al., 2017), highlighting their versatility in capturing the temporal structure inherent to physiological signals.

#### 1.2.3 The attention revolution: transformers in ECG analysis

Transformers have recently surpassed recurrent neural networks (RNNs) in sequence modeling, owing to their self-attention mechanism, which efficiently captures long-range dependencies in parallel (Vaswani et al., 2017). In the context of ECG analysis, several domain-specific adaptations, such as *lead-wise attention, temporal-channel attention*, and *multi-head spatiotemporal transformers*, have been developed to allow models to focus simultaneously on diagnostically relevant waveform segments and leads (Natarajan et al., 2020).

Beyond general classification, attention mechanisms have been integrated into diverse ECG-related applications. These include biometric identification (Hammad et al., 2021; Jyotishi and Dandapat, 2021), CNN-based classification frameworks (Wang et al., 2021; Nejedly et al., 2021; Kuvaev and Khudorozhkov, 2019), fetal ECG extraction from maternal recordings (Mohebbian et al., 2021), and expert-guided attention systems leveraging deep visual interpretability for multi-class disease detection (Huang et al., 2024; Zhang et al., 2020; Tao et al., 2022; Mousavi et al., 2019; Sadad et al., 2023; Lu et al., 2023; Geng et al., 2023; Rafi and Ko, 2022). Attention-based models have also been employed for stress detection from ECG signals (Zhang et al., 2021) and refined self-attention frameworks for disease classification (Tao et al., 2024). Collectively, these advances illustrate the versatility of attention architectures in extracting clinically meaningful information from complex ECG data.

#### 1.2.4 Self-supervised and contrastive pre-training

Self-supervised learning, particularly contrastive learning, has emerged as a powerful approach for ECG representation learning and downstream tasks. Applications include ECG representation and phenotyping (Wei et al., 2022), classification using transformer-based contrastive models (Le et al., 2023; Chen et al., 2021), general ECG modeling (Diamant et al., 2022), stress detection from ECG signals (Rabbani and Khan, 2022), biometric identification through contrastive learning of ECG (Sangha et al., 2024), and anomaly detection frameworks (Li et al., 2022). Several works also explore physiologically inspired augmentations to enhance the robustness and clinical relevance of learned representations.

#### 1.2.5 Hybrid models: the best of all worlds

Many of the best-performing models today are hybrids. For example, CNNs may be used to extract short-term features, followed by transformers for capturing long-range dependencies. Some even combine self-supervised pretraining with supervised fine-tuning on patient-specific data. This modular approach enhances flexibility and makes models more transferable across tasks and institutions.

Together, these developments have led to significant advances in ECG-based diagnostics across a wide range of clinical tasks (Celin and Vasanth, 2018; Jambukia et al., 2015; Malakouti, 2023; Bulbul et al., 2017; Pyakillya et al., 2017; Aziz et al., 2021; Jambukia et al., 2018; Alfaras et al., 2019; Syama et al., 2019), moving the field toward more accurate, data-driven, and scalable cardiovascular assessment.

## 2 Materials and methods

This section is organized into three parts: first, we describe the datasets along with the processing and pre-processing techniques applied; second, we outline the models employed in our study; and third, we detail the experiments conducted to evaluate their performance.

### 2.1 Data processing

#### 2.1.1 Dataset

We evaluated our 12-lead ECG classification framework using the PhysioNet dataset. The first is the PhysioNet ECG-Arrhythmia Database, which comprises 45,152 10-s, resting 12-lead ECG recordings sampled at 500 Hz. ECGs were collected from multiple clinical sites including Chapman University, Shaoxing People's Hospital, and Ningbo First Hospital. Expert clinicians annotated each recording with both rhythm and conduction labels, and the data are stored in standard WFDB format along with demographic metadata. The dataset includes expert-validated annotations for over 60 rhythm and conduction conditions, such as atrial fibrillation, sinus bradycardia, ventricular premature beats, bundle branch blocks, myocardial infarctions in various walls, axis deviations, and repolarization abnormalities, each mapped to corresponding SNOMED CT codes.

The database contains one recording per patient (45,152 patients corresponding to 45,152 recordings), facilitating patient-wise data partitioning without risk of data leakage.

#### 2.1.2 Data preparation

The raw 12-lead ECG recordings were obtained from the PhysioNet database. We curated the data into two distinct datasets based on the type of labels:

- Type 1: ECG records containing a single disease code per patient.
- Type 2: ECG records with one primary disease code and possibly additional comorbidities.

We analyzed three disease categories—Sinus Tachycardia (ST), Sinus Bradycardia (SB), and Sinus Rhythm (SR)—with their SNOMED CT codes and sample sizes listed in Table 1.

**Table 1:** Disease codes and corresponding sample sizes in Type 1 and Type 2 datasets.

Disease	Code	Type 1	Type 2
ST	427084000	2,991	4,987
SB	426177001	4,986	4,996
SR	426783006	4,988	4,984

#### 2.1.3 Superposition, coarse-graining, and invertibility

Let the standard 12-lead ECG recording be represented as a matrix in Equation 1:


$$\begin{array}{l} \text{F}\in {\mathbb{R}}^{T\times 12}, \end{array}$$ (1)

where each column **f**_*l*_ = *f*_*l*_(*t*) corresponds to the temporal signal of lead *l*, for *l* = 1, …, 12. here T is the number of time samples (5,000 for a 10-s recording at 500 Hz).

To construct a unified high-dimensional representation, we transform this multi-channel time series into a two-dimensional spatial–temporal image **I** by superposing the individual leads along an orthogonal spatial basis (Figure 2).

**Figure 2:**
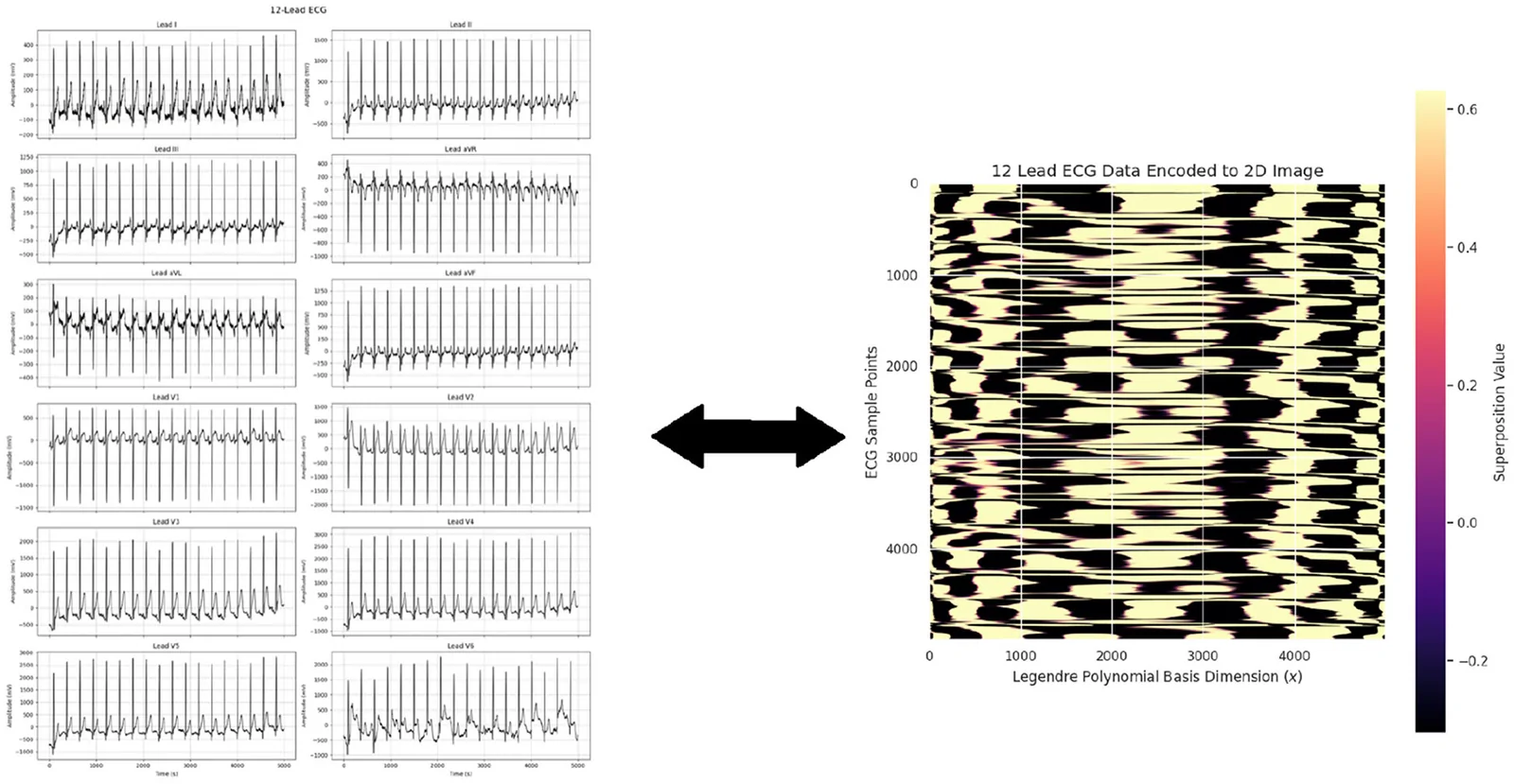
Visualization of the 12-lead ECG encoded into a single image using orthogonal polynomial basis functions. For visualization purposes, the scalar-valued OPI images are displayed using a perceptually uniform pseudocolor colormap. The encoded representation itself remains a single-channel image, which is the representation used throughout all learning experiments.

Each ECG lead is associated with a distinct orthogonal polynomial *P*_*l*_(*x*), drawn primarily from the Legendre family, though Chebyshev polynomials were also explored. These bases possess desirable mathematical properties, including mutual orthogonality and completeness over finite domains, enabling a compact and non-redundant encoding of lead-specific information. Although OPI is named after the underlying family of orthogonal polynomials, the basis functions are normalized in practice, yielding an orthonormal basis that permits exact inversion before coarse-graining. For clarity, we refer to the resulting normalized polynomial basis simply as the orthonormal basis throughout the remainder of this work.

After normalization, the resulting encoding, referred to as Orthogonal Polynomial Imaging (OPI) is defined in Equation 2 as:


$$\begin{array}{l} I\left(x,t\right)=\overset{12}{\underset{l=1}{\sum }}f_{l}\left(t\right)⊗P_{l}\left(x\right), \end{array}$$ (2)

where ⊗ denotes the outer (tensor) product between the temporal signal and its corresponding spatial basis.

Equivalently, in matrix notation, the transformation can be written as in Equation 3:


$$\begin{array}{l} \text{I}=\overset{12}{\underset{l=1}{\sum }}{\text{f}}_{l}{\text{p}}_{l}^{T}, \end{array}$$ (3)

where **p**_*l*_ is the discretized and normalized spatial basis vector corresponding to *P*_*l*_(*x*).

This formulation makes explicit the superpositional structure of the encoding: each lead contributes an independent rank-one component, and their summation yields a coarse-grained spatial–temporal image that preserves both temporal dynamics and inter-lead distinctions.

##### 2.1.3.1 Inverse framework and information preservation

To support the claim that the proposed transformation is information-preserving, we define an explicit inverse operation. Owing to the orthonormality of the spatial basis vectors ${\left\{{\text{p}}_{l}\right\}}_{l=1}^{12}$, each original ECG lead can be exactly recovered by projecting the image **I** onto the corresponding basis vector, as defined in Equation 4:


$$\begin{array}{l} {\text{f}}_{l}=\text{I}{\text{p}}_{l}, \end{array}$$ (4)

for *l* = 1, …, 12.

This inverse formulation demonstrates that no information is lost during the encoding process; rather, the transformation constitutes a structured reparameterization of the original signal. Since the normalized polynomial basis forms an orthonormal basis, OPI provides a bijective mapping between the multi-lead ECG time series and its two-dimensional representation prior to coarse-graining, enabling downstream learning models to operate on a unified spatial structure while retaining full access to lead-wise temporal information.

The resulting function *I*(*x, t*) forms a 5,000 × 5,000 superposition image that preserves information from all leads while compactly fusing the multi-lead signals into a single higher-dimensional structure.

To reduce computational cost and facilitate efficient training, these superposition images were coarse-grained using an averaging operation over non-overlapping blocks. Each pixel in a coarse-grained image corresponds to the mean value of the corresponding block in the original superposition matrix, given by Equation 5:


$$\begin{array}{l} I_{\text{cg}}\left[i,j\right]=\frac{1}{cg^{2}}\overset{\left(i+1\right)\cdot cg}{\underset{p=i\cdot cg}{\sum }}\overset{\left(j+1\right)\cdot cg}{\underset{q=j\cdot cg}{\sum }}I\left[p,q\right]. \end{array}$$ (5)

In practice, we used block sizes of 50 × 50 and 100 × 100, producing final images of size 100 × 100 and 50 × 50, respectively.

Before applying the superposition procedure, each individual 12-lead ECG sample, represented as a matrix of size 5,000 × 12, is independently normalized and standardized using fit_transform(each sample). All normalization parameters are computed on a per-sample and per-lead basis, without using statistics from other samples or from the test set, thereby preventing information leakage.

For each ECG sample, we applied fit_transform from StandardScaler in scikit-learn, which independently standardizes each lead by subtracting its temporal mean and scaling it to unit variance.

This preprocessing step ensures uniform scaling across leads within each ECG recording and allows each lead to contribute comparably to the final image representation.

This approach effectively captures inter-lead relationships while maintaining salient temporal features of the ECG, resulting in compact 2D images that are suitable as inputs for both fully connected and convolutional neural networks.

The average preprocessing time was approximately 222.55s (about 3.71 min) per batch of 500 ECG records.

#### 2.1.4 Splits

For all experiments, data are partitioned using a patient-wise 70–15–15 train-validation-test split. Since each patient in the PhysioNet ECG Arrhythmia database contributes exactly one recording, this ensures no subject appears in multiple sets. The splits are implemented using five different random seeds (random_state ∈ {40, 41, 42, 43, 44}) to ensure reproducibility.

### 2.2 Models

#### 2.2.1 Feed forward neural networks for single lead

We employed a fully connected feed-forward neural network trained on the single-lead ECG data (5,000).

The network architecture consisted of five hidden layers:


$$\begin{array}{l} \text{Input: }5,000\\ \text{Hidden Layers: }1,000\to 250\to 100\to 30\to 10\\ \text{Output: }3 \end{array}$$

Each hidden layer was followed by a ReLU activation function. To reduce overfitting, dropout layers with a dropout rate of 0.3 were applied after each hidden layer. Batch normalization was not employed.

The output layer consisted of three neurons corresponding to the target classes. Since the categorical cross-entropy loss function implicitly applies the Softmax activation, no explicit activation function was used in the final layer.

The model was trained using the Adam optimizer with a learning rate of 1 × 10^−3^ and a weight decay of 1 × 10^−4^ to further prevent overfitting. The categorical cross-entropy loss function was used for multi-class classification. The batch size was set to 32, and training was carried out for a maximum of 25 epochs.

To improve convergence, a learning rate scheduler was employed, which reduced the learning rate by a factor of 0.5 if the validation loss did not improve for two consecutive epochs. Early stopping with a patience of seven epochs was applied to terminate training when no improvement in validation accuracy was observed.

Early stopping often terminated training before reaching the maximum number of 25 epochs. The best validation accuracy achieved during training was recorded and used for final evaluation.

All experiments were implemented in PyTorch, and computations were carried out on an NVIDIA GeForce RTX 3050 GPU with CUDA support, otherwise defaulting to a CPU environment.

#### 2.2.2 Convolutional neural networks

To further enhance the model's ability to capture spatial hierarchies and complex local patterns inherent in medical image data, we employed a unified Convolutional Neural Network (CNN) architecture, referred to as *SmallCNN*. Unlike fully connected networks, CNNs exploit spatial locality through convolutional kernels, enabling the model to learn translationally invariant features from the encoded ECG images.

To facilitate experiments across multiple spatial resolutions, the architecture includes an Adaptive Average Pooling layer. This allows the network to process inputs of varying sizes (e.g., 100 × 100 or 50 × 50) and produce a fixed-size feature representation for the classification head without changing the model parameters.

Reproducibility and Data Integrity: To ensure the scientific validity of our results, the following experimental protocols were strictly followed:

- Patient-Independent Splits: All datasets were split into training, validation, and test sets using a strictly patient-wise strategy with a 70–15–15 split. No data from any single subject appears in more than one set, preventing identity-based leakage.
- Stratification and Seed: Splits were stratified to maintain the original class distribution of ST, SB, and SR. All experiments were conducted with five different random seeds (40, 41, 42, 43, 44) for deterministic weight initialization and reproducibility.

The detailed architecture of the *SmallCNN* is summarized in Table 2:

**Table 2:** Specifications of the unified SmallCNN architecture.

Layer (type)	Output dimension	Kernel/stride	Activation/dropout
Input Image	*H* × *W* × 1	–	–
Conv2d (1)	*H* × *W* × 32	3 × 3, *p* = 1	BatchNorm + ReLU
MaxPool2d	*H*/2 × *W*/2 × 32	2 × 2	Dropout (*p* = 0.25)
Conv2d (2)	*H*/2 × *W*/2 × 64	3 × 3, *p* = 1	BatchNorm + ReLU
MaxPool2d	*H*/4 × *W*/4 × 64	2 × 2	–
Conv2d (3)	*H*/4 × *W*/4 × 128	3 × 3, *p* = 1	BatchNorm + ReLU
MaxPool2d	*H*/8 × *W*/8 × 128	2 × 2	–
AdaptivePool	4 × 4 × 128	–	Fixed output
Flatten	2,048	–	Dropout (*p* = 0.5)
Linear (dense 1)	512	–	ReLU
Linear (output)	3 (ST, SB, SR)	–	Dropout (*p* = 0.5)

The model was trained using the Adam optimizer with two initial learning rates (1 × 10^−3^ and 2 × 10^−3^) for the *SmallCNN* model. Apart from the initial learning rate, all training hyperparameters were identical across experiments, including a weight decay of 1 × 10^−4^, categorical cross-entropy loss, and a training duration of 25 epochs. Early stopping with a patience of 7 epochs and a learning rate scheduler (ReduceLROnPlateau, factor 0.5, patience 2) were employed to prevent overfitting and improve generalization.

#### 2.2.3 Vanilla transformer

To evaluate the OPI framework against a high-capacity sequence-modeling baseline, we implemented a *Vanilla Transformer* architecture designed for multi-lead ECG classification. This baseline operates directly on the raw 1D temporal signals, allowing for a comparison between attention-based sequence modeling and our proposed 2D vision-based embedding.

Architecture and Tokenization: The input ECG signal **X** ∈ ℝ^12×5,000^ is processed using a patch-based tokenization approach. The sequence length (*L* = 5,000) is divided into *N* = 100 non-overlapping patches, each of size *P* = 50 samples. To capture inter-lead correlations within each time-step, patches from all 12 leads are concatenated, resulting in a flattened vector of dimension 12 × 50 = 600 per patch.

These vectors are projected into a *d*_*model*_ = 128 latent space using a linear embedding layer. A learnable class (CLS) token is prepended to the sequence, and fixed sinusoidal positional encodings are added to the embeddings to retain temporal order. The core of the model consists of 4 Transformer encoder layers, each utilizing 8 attention heads and a feedforward network dimension of 512 with GELU activation.

Training Protocol: The Transformer was trained following the same data integrity standards as the OPI-CNN models:

- Data Splits: A strictly patient-independent 70–15–15 split was maintained to prevent data leakage.
- Optimizer: Adam optimizer was used with an initial learning rate of 1 × 10^−4^ and a weight decay of 1 × 10^−4^.
- Regularization: A dropout rate of 0.1 was applied within the encoder layers, and 0.5 within the final classification head to mitigate overfitting.

The configuration of the VanillaTransformerECG is summarized in Table 3:

**Table 3:** Hyperparameters for the baseline vanilla transformer.

Hyperparameter	Value
Patch size (*P*)	50 samples
Number of patches (*N*)	100
Embedding dim (*d*_*model*_)	128
Attention heads (*nhead*)	8
Encoder layers	4
Feedforward dim	512
Activation function	GELU
Dropout	0.1 (encoder), 0.5 (head)

#### 2.2.4 ResNet model

To evaluate whether the OPI framework provides superior feature representation compared to standard lead-by-time matrices, we implemented a modified *ResNet-18* baseline, hereafter referred to as ECGResNet. This model processes the raw 12-lead ECG signal as a single-channel 2D image of dimension 12 × 5,000 without any orthogonal embedding.

Architecture and Adaptation: We adapted the standard ResNet-18 architecture to handle grayscale medical data:

- Input Layer: The initial convolutional layer was modified to accept 1 input channel instead of the standard 3 (RGB). To leverage transfer learning, the weights were initialized by averaging the pretrained ImageNet weights across the input channels.
- Spatial Hierarchy: The model utilizes the standard four-stage residual blocks, followed by global average pooling and a final dense layer for 3-class classification.

This baseline tests the 2D approach, allowing us to quantify the performance gain achieved by projecting signals onto an orthogonal spatial manifold (OPI) vs. using a raw data grid.

#### 2.2.5 SelfEEG ResNet1D baseline

To provide a strong temporal baseline operating directly on the raw signal, we trained the ResNet1D architecture from the SelfEEG library (Del Pup et al., 2024), hereafter referred to as SelfEEGResNet1D. This model is a residual 1D convolutional neural network designed for time-series data and processes the raw ECG waveform without any spatial encoding. The network takes as input the multi-lead or single-lead ECG signal (either 12 × 5,000 for all leads or 1 × 5,000 for Lead 1 alone), where convolutions operate exclusively along the temporal dimension to learn hierarchical temporal representations.

This baseline evaluates a purely temporal convolutional architecture trained directly on the raw ECG signals, providing a strong 1D CNN reference for comparison with both the 2D ResNet baseline and the proposed OPI-based representation. A single-lead variant using only Lead 1 was also trained to assess the contribution of multi-lead information within the same architectural framework.

Architecture:

- Input Stem: An initial convolutional layer with kernel size 7 followed by batch normalization and ReLU activation expands the feature representation while preserving the temporal resolution.
- Residual Stages: Four sequential residual stages with channel progression 32 → 64 → 128 → 256. Each stage contains two basic residual blocks (He et al., 2016) with kernel size 7. Temporal downsampling is introduced via strided convolutions at the transitions between stages.
- Classification Head: A global average pooling layer aggregates temporal features, followed by a fully connected layer producing predictions for the three ECG classes.

### 2.3 Experiments

We conducted experiments on the two dataset configurations Type 1 and Type 2 mentioned in Section 2.1.2. Across both dataset types, we evaluated three categories of input representations: (1) orthogonal polynomial image encodings of multi-lead ECGs, (2) raw multi-lead ECG signals, and (3) raw single-lead ECG signals. Neural network architectures were selected based on the input modality, as summarized below.

#### 2.3.1 Image-based encodings

Using the superposition and coarse-graining framework, the 12-lead ECG signals were encoded into two-dimensional images via orthogonal polynomials (Legendre and Chebyshev).

- CNN on 100 × 100 images: Trained separately on Legendre, and Chebyshev.
- CNN on 50 × 50 images: Trained on Legendre encoding.

#### 2.3.2 Single-lead signals

To provide a baseline independent of polynomial encoding, we also trained a fully connected neural network (FFNN) directly on the raw ECG waveform from the first lead. Each input sample consisted of a one-dimensional vector of length 5,000, corresponding to the full temporal sequence.

#### 2.3.3 ResNet

To benchmark against standard 2D image-based approaches, we trained a ECGResNet model on the raw lead-by-time ECG matrix of size 12 × 5,000 on both Type 1 and Type 2 datasets. In addition, we trained a non-pretrained variant of the same architecture (randomly initialized weights) to isolate the effect of transfer learning from ImageNet pretraining.

#### 2.3.4 Vanilla transformer

To benchmark against high-capacity sequence models, we trained the Vanilla Transformer model on the raw 12-lead ECG signals on both Type 1 and Type 2 datasets.

#### 2.3.5 ResNet1D

To benchmark against strong temporal convolutional architectures, we trained a ResNet1D model from the SelfEEG library on the raw 12-lead ECG signals. The model operates directly on the temporal dimension and processes the input as a multichannel time-series signal, providing a strong 1D CNN baseline for both Type 1 and Type 2 datasets. A single-lead variant using only Lead 1 (1 × 5,000) was also trained to evaluate the contribution of multi-lead information within the same 1D residual framework.

#### 2.3.6 Training configuration

##### 2.3.6.1 Reproducibility

To ensure robustness and reproducibility across runs, we conducted all experiments across five independent trials using different random seeds (40, 41, 42, 43, 44). For each trial, we set the respective random seeds for NumPy and PyTorch and enforced deterministic GPU execution by disabling non-deterministic cuDNN optimizations.

Common Hyperparameter (All Models):

- Criterion: CrossEntropyLoss()
- Optimizer: Adam (weight_decay = 1 × 10^−4^)
- Scheduler: ReduceLROnPlateau(mode = “min,” factor = 0.5, patience = 2)
- Training: maximum of 25 epochs with early stopping (patience = 7)
- Batch Size: 32

The model-specific configurations and learning rates across all evaluated architectures are summarized in Table 4.

**Table 4:** Model-specific configuration (only differences shown).

Model name	Architecture	Learning rate
2D CNN - Legendre (100×100 Image)		
2d_legendre_model_typ1	SmallCNN 2.2.2	1 × 10^−3^
2d_legendre_model_typ2	SmallCNN 2.2.2	2 × 10^−3^
2D CNN - Legendre (50×50 Image)		
2d_legendre_basis50_typ1	SmallCNN 2.2.2	1 × 10^−3^
2d_legendre_basis50_typ2	SmallCNN 2.2.2	1 × 10^−3^
FFNN - Single Lead (first lead)		
1d_ffnn_singlelead_typ1	Model_ffnn 2.2.1 (single lead input)	1 × 10^−3^
1d_ffnn_singlelead_typ2	Model_ffnn 2.2.1 (single lead input)	1 × 10^−3^
2D ResNet Models (pretrained=True)		
2d_resnet_typ1	ECGResNet 2.2.4 (pretrained=True)	1 × 10^−3^
2d_resnet_typ2	ECGResNet 2.2.4 (pretrained=True)	1 × 10^−3^
2D ResNet Models (pretrained=False)		
2d_resnet_typ1	ECGResNet 2.2.4 (pretrained=False)	1 × 10^−3^
2d_resnet_typ2	ECGResNet 2.2.4 (pretrained=False)	1 × 10^−3^
Transformer Models		
vanilla_transformer_typ1	VanillaTransformerECG 2.2.3	1 × 10^−4^
vanilla_transformer_typ2	VanillaTransformerECG 2.2.3	1 × 10^−4^
2D CNN - Chebyshev		
2d_cheb_typ1	SmallCNN 2.2.2	1 × 10^−3^
2d_cheb_typ2	SmallCNN 2.2.2	2 × 10^−3^
1D-ResNet Models		
resnet1d_typ1	SelfEEGResNet1D 2.2.5	1 × 10^−3^
resnet1d_typ2	SelfEEGResNet1D 2.2.5	1 × 10^−3^
1D-ResNet Models - Single Lead (Lead 1)		
resnet1d_singlelead_typ1	SelfEEGResNet1D 2.2.5 (single lead input)	1 × 10^−3^
resnet1d_singlelead_typ2	SelfEEGResNet1D 2.2.5 (single lead input)	1 × 10^−3^

## 3 Results

We report results from eight experimental setups using the models described in Section 2. We first benchmark our OPI-encoded ECG representations, smoothed and resized to 100 × 100, using Convolutional Neural Networks to assess baseline performance. To evaluate the robustness of the proposed encoding, we repeat the analysis with more aggressively downsampled representations of size 50 × 50. We then compare these results against six established baselines: a pretrained 2D ResNet, a non-pretrained 2D ResNet, a 1D ResNet (SelfEEG), a single-lead 1D ResNet (SelfEEG), a vanilla 1D Transformer, and a feedforward neural network trained on a single ECG lead. All experiments are conducted on two distinct datasets, referred to as Type 1 and Type 2, as described in Section 2.

### 3.1 Benchmarking OPI : data Type 1 encoded using Legendre polynomials

All 12-lead ECG signals of Data Type 1 were first converted into images of size 5,000 × 5,000 using Legendre Polynomial encoding, which were then down-sampled to 100 × 100 and 50 × 50 resolutions using coarse-graining (see Section 2.1.3). The CNN was trained on both 100 × 100 and 50 × 50 images to assess the effect of resolution. The dataset comprised 2991 ST, 4986 SB, and 4988 SR samples, with a 70–15–15 split for training-validation-test.

#### 3.1.1 Benchmarking OPI using CNN

We begin by employing a compact Convolutional Neural Network (CNN) on the encoded ECG images. This architecture effectively extracts hierarchical spatial features and captures local structural patterns inherent in the encoded representations, enabling accurate classification across the three target classes.

The CNN exhibited rapid and stable convergence (Figure 3). The confusion matrix in Figure 4 indicates strong per-class performance, with correct classification rates of *98.75%* ± *0.46%* for ST, *99.81%* ± *0.14%* for SB, and *97.25%* ± *0.41%* for SR. As summarized in Table 5, the CNN achieved an overall accuracy of *98.58%* ± *0.13%*, with macro- and weighted-average precision, recall, and F1-scores of *0.985*. The ROC curves in Supplementary Figure 1a further confirm excellent discriminative capability, with AUC values of *0.9988* ± *0.0001* (ST), *0.9997* ± *0.0000* (SB), and *0.9992* ± *0.0001* (SR), with a micro-averaged AUC of *0.9993* ± *0.0000* and a macro-averaged AUC of *0.9992*. Finally, the micro-averaged average precision score reached *0.9989* ± *0.0001* (Supplementary Figure 1b), demonstrating superior performance compared to the FFNN baseline.

**Figure 3:**
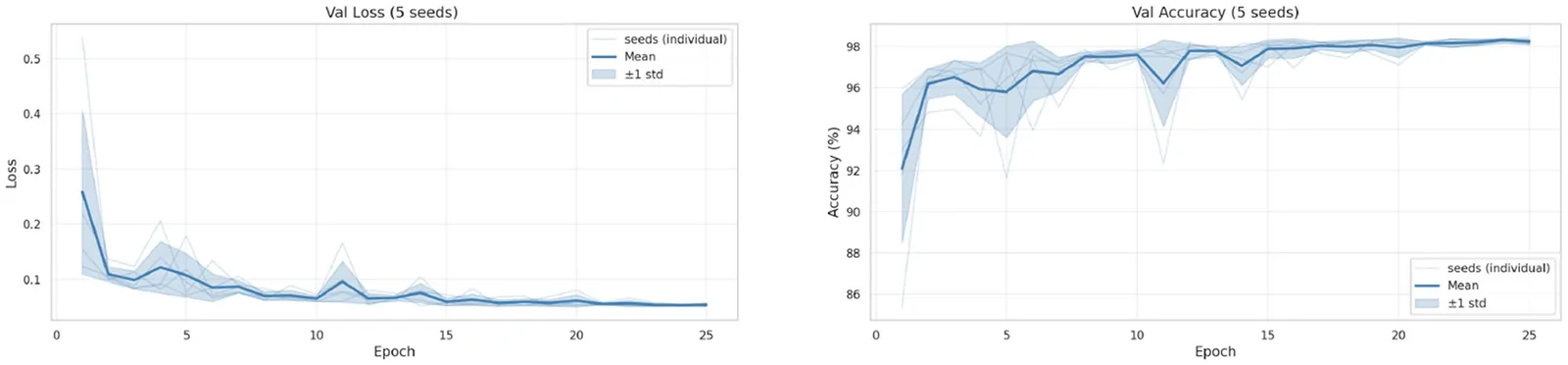
Validation losses **(Left)** and accuracies **(Right)** for the CNN over 25 epochs on 100 × 100 Legendre images.

**Figure 4:**
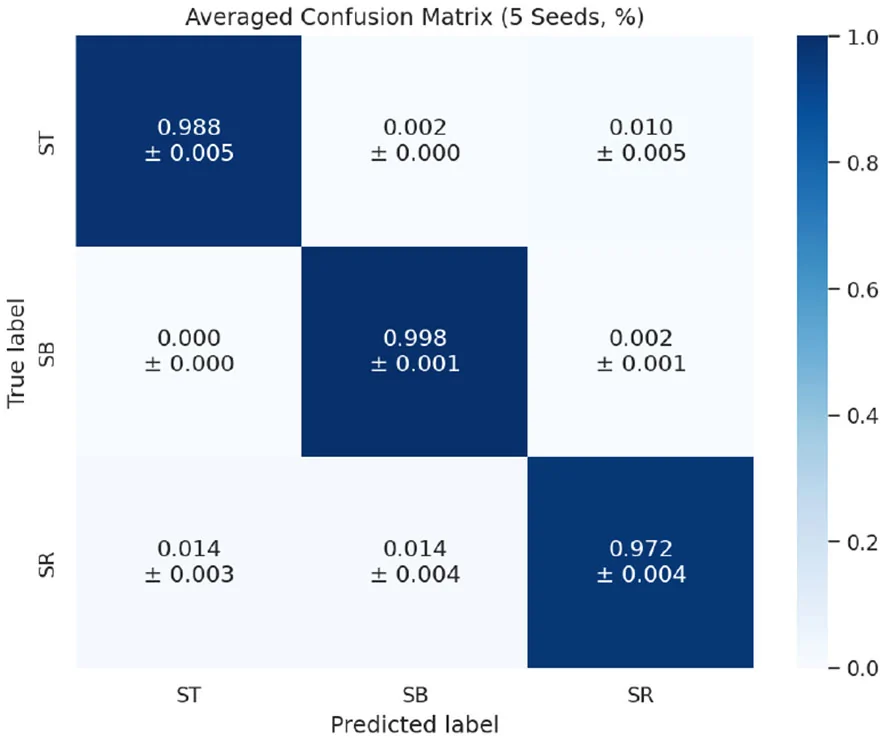
Confusion matrix for CNN classification on Data Type 1.

**Table 5:** Classification report for the CNN model on Data Type 1.

Class	Precision	Recall	F1-score	Support
ST	0.9771 ± 0.0048	0.9875 ± 0.0046	0.9823 ± 0.0025	449
SB	0.9852 ± 0.0035	0.9981 ± 0.0014	0.9916 ± 0.0012	748
SR	0.9918 ± 0.0025	0.9725 ± 0.0041	0.9820 ± 0.0016	748
Accuracy			0.9858 ± 0.0013	1,945
Macro Avg	0.9847 ± 0.0014	0.9860 ± 0.0014	0.9853 ± 0.0015	1,945
Weighted Avg	0.9859 ± 0.0013	0.9858 ± 0.0013	0.9858 ± 0.0013	1,945

#### 3.1.2 Benchmarking OPI for reduced image size using CNN

To investigate the impact of increased image smoothing and reduced spatial resolution, we trained a CNN model on ECG images downsampled to a size of 50 × 50. The same CNN architecture used for the 100 × 100 images was adapted to this smaller input size. The primary motivation was to evaluate whether the diagnostic performance remains robust when finer-grained details are smoothed out due to aggressive compression.

The CNN demonstrated stable convergence (Figure 5). The confusion matrix in Figure 6 shows per-class performance, with high recall across all classes, particularly for SB. The ROC analysis in Supplementary Figure 2a confirms strong discriminative capability, with AUC values of *0.9966* ± *0.0002* for ST, *0.9993* ± *0.0001* for SB, and *0.9957* ± *0.0005* for SR, as well as micro- and macro-averaged AUCs of *0.9979* ± *0.0002* and *0.9972*, respectively. Table 6 summarizes the precision, recall, and F1-scores. The CNN achieved an overall test accuracy of *97.33%* ± *0.18%*, with macro- and weighted-average F1-scores of *0.9718* ± *0.0016* and *0.9718* ± *0.0016*, indicating balanced performance despite the reduced image resolution. Finally, the micro-averaged average precision score reached *0.9962* ± *0.0004* (Supplementary Figure 2b).

**Figure 5:**
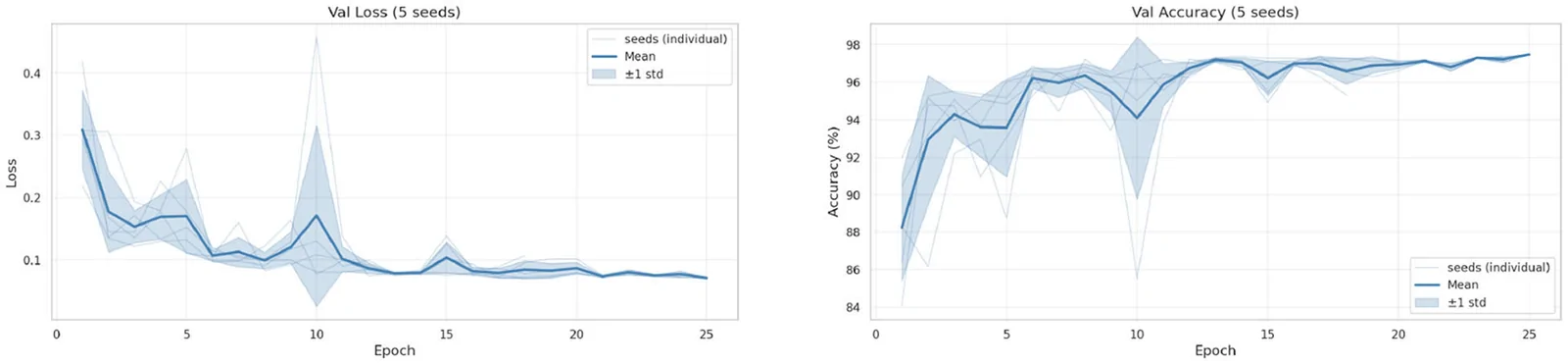
Validation losses **(Left)** and accuracies **(Right)** for the CNN on 50 × 50 Legendre images over 25 epochs.

**Figure 6:**
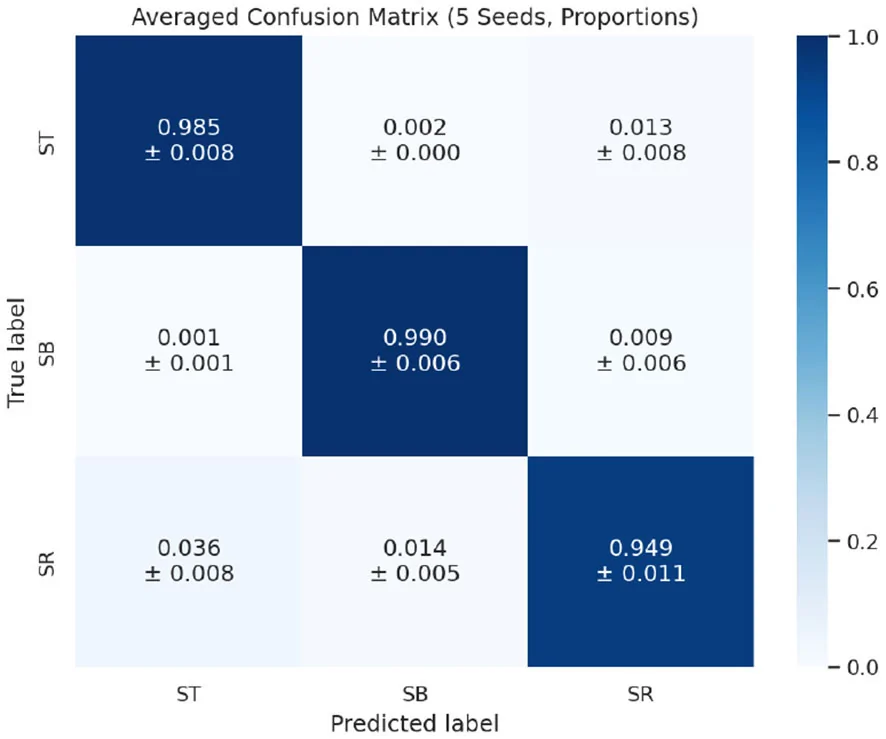
Confusion matrix for CNN classification on 50 × 50 Legendre images for Data Type 1.

**Table 6:** Classification report for the CNN model on 50 × 50 Legendre-encoded images (Data Type 1).

Class	Precision	Recall	F1-score	Support
ST	0.9490 ± 0.0040	0.9850 ± 0.0080	0.9665 ± 0.0040	449
SB	0.9788 ± 0.0048	0.9900 ± 0.0060	0.9844 ± 0.0030	748
SR	0.9647 ± 0.0084	0.9490 ± 0.0110	0.9568 ± 0.0050	748
Accuracy			0.9733 ± 0.0018	1,945
Macro Avg	0.9642 ± 0.0050	0.9747 ± 0.0050	0.9718 ± 0.0016	1,945
Weighted Avg	0.9642 ± 0.0050	0.9747 ± 0.0050	0.9718 ± 0.0016	1,945

### 3.2 Data Type 2 encoded using Legendre polynomials

We next report results on Data Type 2, where each ECG record includes a primary disease label and potential comorbidities. This setting introduces higher variability compared to Type 1. The same pre-processing pipeline was applied: 12-lead ECGs were Legendre-encoded into 5,000 × 5,000 images and down-sampled to 100 × 100 and 50 × 50. The dataset consisted of *4987* ST, *4996* SB, and *4984* SR samples, split 70–15–15 for training-validation-test.

#### 3.2.1 Benchmarking OPI using CNN

For Data Type 2, the CNN exhibited stable convergence (Figure 7) and strong generalization. The confusion matrix (Figure 8) indicates balanced performance across classes, with recall values of *97.70%* ± *0.70%* for ST, *98.30%* ± *0.40%* for SB, and *95.60%* ± *0.70%* for SR, confirming SB as the most separable rhythm. Table 7 reports an overall accuracy of *97.19%* ± *0.29%*, with macro- and weighted-average precision, recall, and F1-scores all equal to *0.9718* ± *0.0029*. The ROC analysis (Supplementary Figure 3a) further confirms strong discriminative capability, with macro-averaged AUC and micro-averaged AP of *0.9970* and *0.9933* ± *0.0008*, respectively.

**Figure 7:**
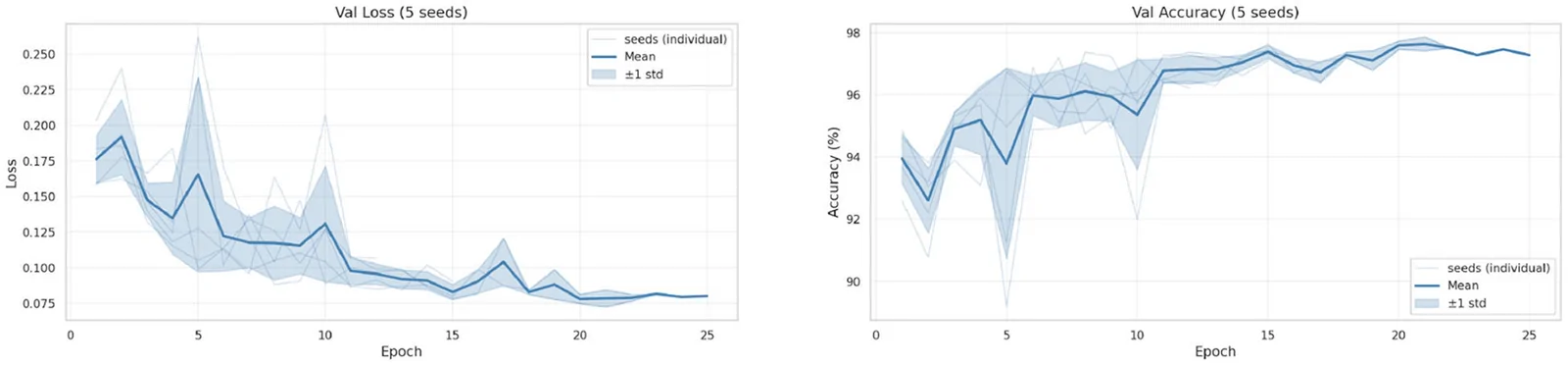
Validation losses **(Left)** and accuracies **(Right)** for the CNN over 25 epochs on Data Type 2.

**Figure 8:**
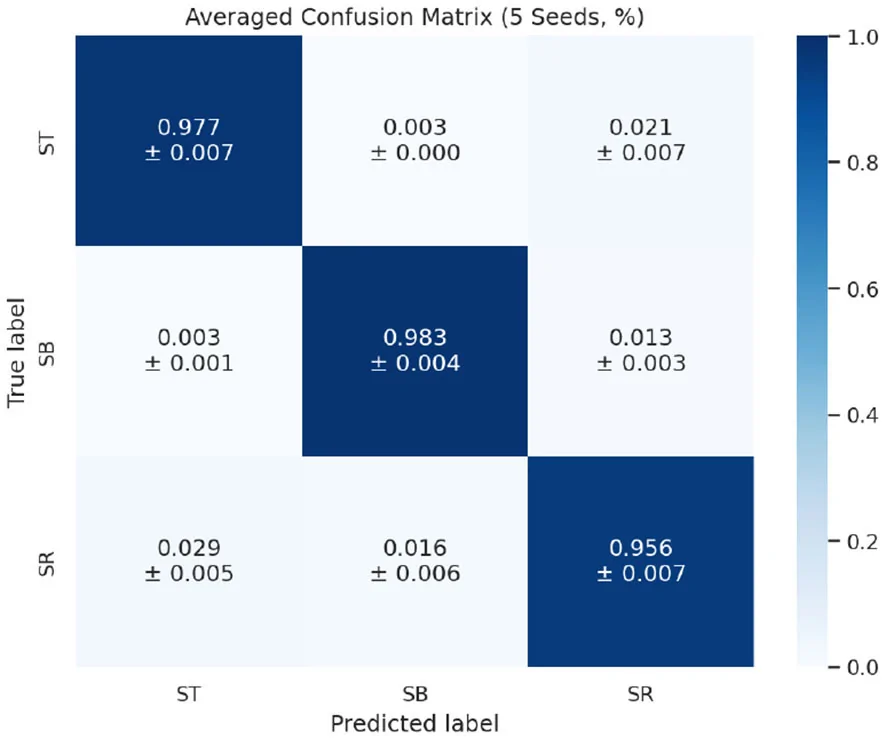
Confusion matrix for CNN classification on Data Type 2.

**Table 7:** Classification report for the CNN model on Data Type 2.

Class	Precision	Recall	F1-score	Support
ST	0.9680 ± 0.0049	0.9770 ± 0.0070	0.9725 ± 0.0035	748
SB	0.9810 ± 0.0040	0.9830 ± 0.0040	0.9820 ± 0.0025	750
SR	0.9660 ± 0.0045	0.9560 ± 0.0070	0.9610 ± 0.0030	748
Accuracy			0.9719 ± 0.0029	2,246
Macro Avg	0.9717 ± 0.0029	0.9720 ± 0.0029	0.9718 ± 0.0029	2,246
Weighted Avg	0.9717 ± 0.0029	0.9720 ± 0.0029	0.9718 ± 0.0029	2,246

#### 3.2.2 Benchmarking OPI for reduced image size using CNN

To evaluate the impact of increased smoothing and reduced spatial resolution on Data Type 2, a CNN was trained on ECG images downsampled to *50* × *50*. The same architecture used for *100* × *100* inputs was adapted to the smaller resolution to assess whether diagnostic performance remains reliable under image compression.

The CNN showed stable convergence on Data Type 2 (Figure 9). The confusion matrix in Figure 10 indicates strong per-class performance, with recall values of *98.02%* ± *0.50%* for ST, *97.20%* ± *0.76%* for SB, and *93.96%* ± *0.65%* for SR. Table 8 reports an overall accuracy of *96.39%* ± *0.23%*, with macro- and weighted-average F1-scores of approximately *0.964* ± *0.002*. The ROC analysis in Supplementary Figure 4a confirms good separability, with AUC values of *0.9949* ± *0.0004* (ST), *0.9972* ± *0.0001* (SB), and *0.9928* ± *0.0004* (SR), with a micro-averaged AUC of *0.9950* and a macro-averaged AUC of *0.9950*. Finally, the micro-averaged average precision score reached *0.9908* ± *0.0008*, as shown in Supplementary Figure 4b.

**Figure 9:**
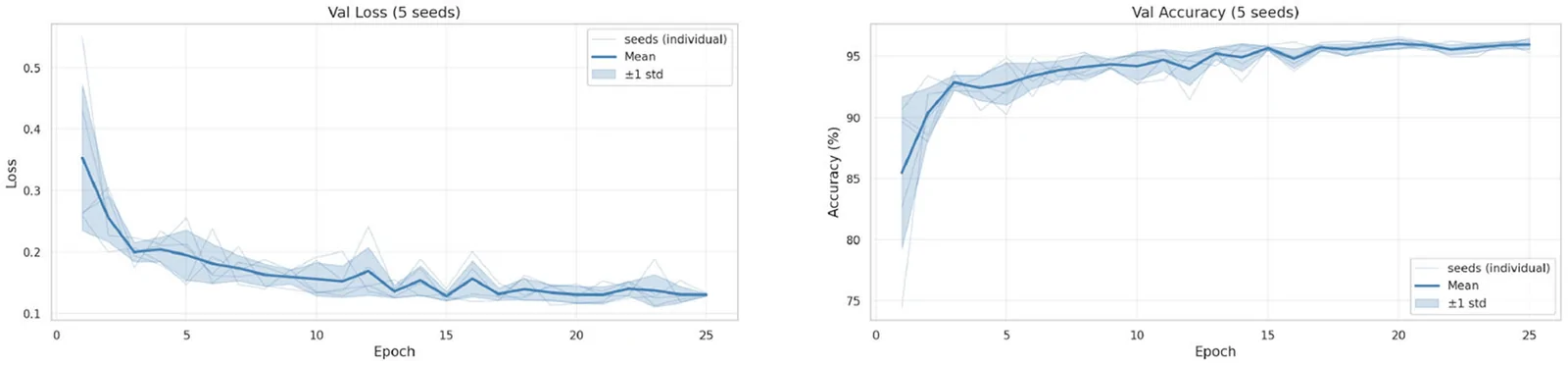
Validation losses **(Left)** and accuracies **(Right)** for the CNN on 50 × 50 Legendre images over 25 epochs for Data Type 2.

**Figure 10:**
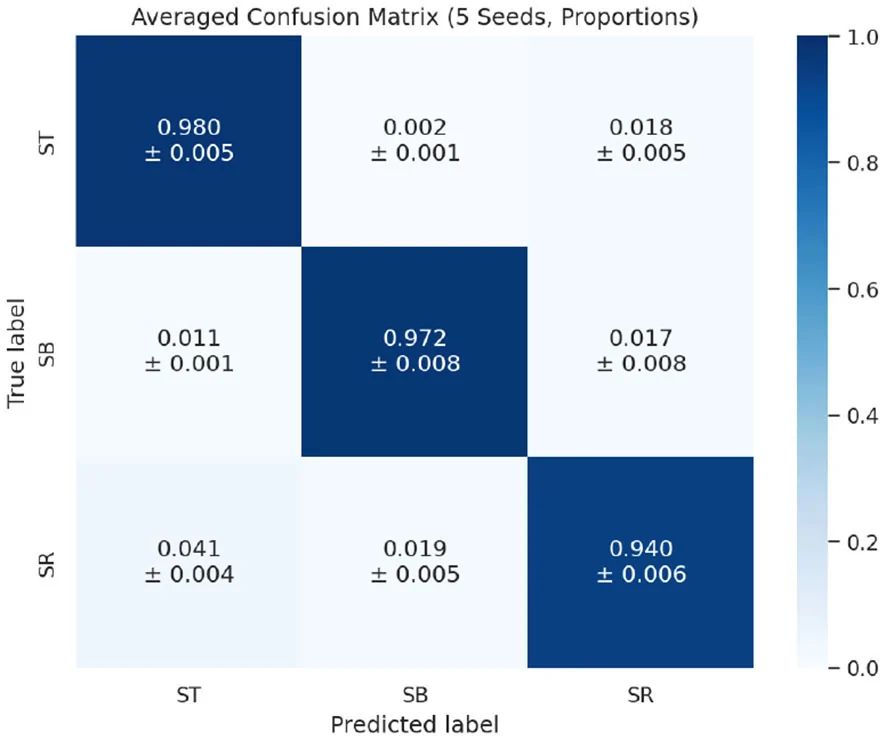
Confusion matrix for CNN classification on 50 × 50 Legendre images for Data Type 2.

**Table 8:** Classification report for the CNN model on 50 × 50 Legendre images for Data Type 2.

Class	Precision	Recall	F1-score	Support
ST	0.9490 ± 0.0040	0.9802 ± 0.0050	0.9644 ± 0.0019	748
SB	0.9788 ± 0.0048	0.9720 ± 0.0076	0.9754 ± 0.0026	750
SR	0.9647 ± 0.0084	0.9396 ± 0.0065	0.9519 ± 0.0030	748
Accuracy			0.9639 ± 0.0023	2,246
Macro Avg	0.9642 ± 0.0023	0.9639 ± 0.0023	0.9639 ± 0.0023	2,246
Weighted Avg	0.9642 ± 0.0023	0.9639 ± 0.0023	0.9639 ± 0.0023	2,246

### 3.3 Baseline 1 : ResNet

We use a modified ResNet-18 architecture (described in Section 2.2.4), denoted as ECGResNet, as the baseline model for several reasons:

1. ResNet architectures are well-established benchmarks and have demonstrated strong performance on ECG classification tasks.
2. A pretrained ResNet-18 provides a robust signal-domain reference and serves as a widely adopted baseline for learning directly from raw ECG signals.
3. Its proven effectiveness enables a fair evaluation: if orthogonal-polynomial imaging (OPI) representations achieve competitive or superior performance, this validates their utility against a strong baseline.
4. The 12-lead ECG is treated as a 2D signal with leads and time forming the two spatial dimensions, and the architecture accepts input of shape (12, 5,000).

The motivation is to compare learning directly from raw ECG signals with learning from orthogonal-polynomial imaging (OPI) representations of the same data, thereby isolating the impact of the input representation on classification performance.

#### 3.3.1 Normalization

Normalization statistics are computed using the training set only to avoid data leakage. Specifically, the mean and standard deviation are calculated per ECG lead across all training samples and time points. A small constant is applied for numerical stability. The same statistics are then used to normalize the training, validation, and test sets according to Equations 6, 7:


$$\begin{array}{l} \mu =\text{mean}\left(X_{\text{train}}\right),\;\sigma =\max\left(\text{std}\left(X_{\text{train}}\right),\epsilon \right), \end{array}$$ (6)

$$\begin{array}{l} X=\frac{X-\mu }{\sigma },\;\epsilon =10^{-6}. \end{array}$$ (7)

#### 3.3.2 Data Type 1

The 2D ResNet (Type 1 configuration) achieves excellent performance on the three-class ECG classification task, substantially outperforming the vanilla Transformer baselines. Leveraging pretrained weights and residual connections, the model converges rapidly (Figure 11), achieving an average test accuracy of 98.09% ± 0.39% across the five seeds (Table 9). A detailed evaluation confirms the robustness and near-ceiling performance of the model across all classes (Figure 12). The macro-averaged precision, recall, and F1-score reach 0.980, 0.981, and 0.981, respectively, with particularly strong performance for the SB class (F1-score 0.988 ± 0.003). Even the more challenging ST and SR classes achieve F1-scores exceeding 0.97. Discriminative ability is exceptionally high, as reflected by ROC–AUC values above 0.997 for all classes and micro- and macro-averaged AUCs of *0.9989* and *0.9986*, respectively. Overall, these results demonstrate that a pretrained 2D ResNet provides highly expressive representations for ECG signals, capturing both local and long-range temporal patterns with near-optimal classification performance.

**Figure 11:**
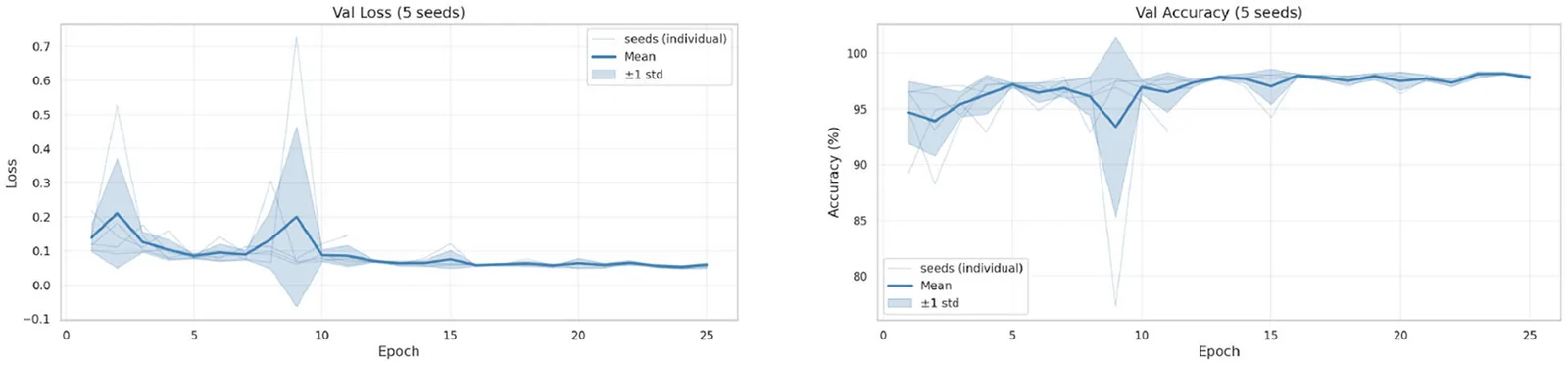
Validation losses **(Left)** and accuracies **(Right)** for the ResNet model on Data Type 1.

**Table 9:** Classification report for the ResNet model applied to 12-lead ECG signals (Data Type 1).

Class	Precision	Recall	F1-score	Support
ST	0.9720 ± 0.0106	0.9844 ± 0.0055	0.9781 ± 0.0040	449
SB	0.9856 ± 0.0022	0.9898 ± 0.0051	0.9877 ± 0.0026	748
SR	0.9817 ± 0.0069	0.9698 ± 0.0079	0.9757 ± 0.0046	748
Accuracy			0.9809 ± 0.0039	
Macro Avg	0.9798 ± 0.0041	0.9813 ± 0.0030	0.9805 ± 0.0039	1,945
Weighted Avg	0.9810 ± 0.0034	0.9809 ± 0.0039	0.9809 ± 0.0039	1,945

**Figure 12:**
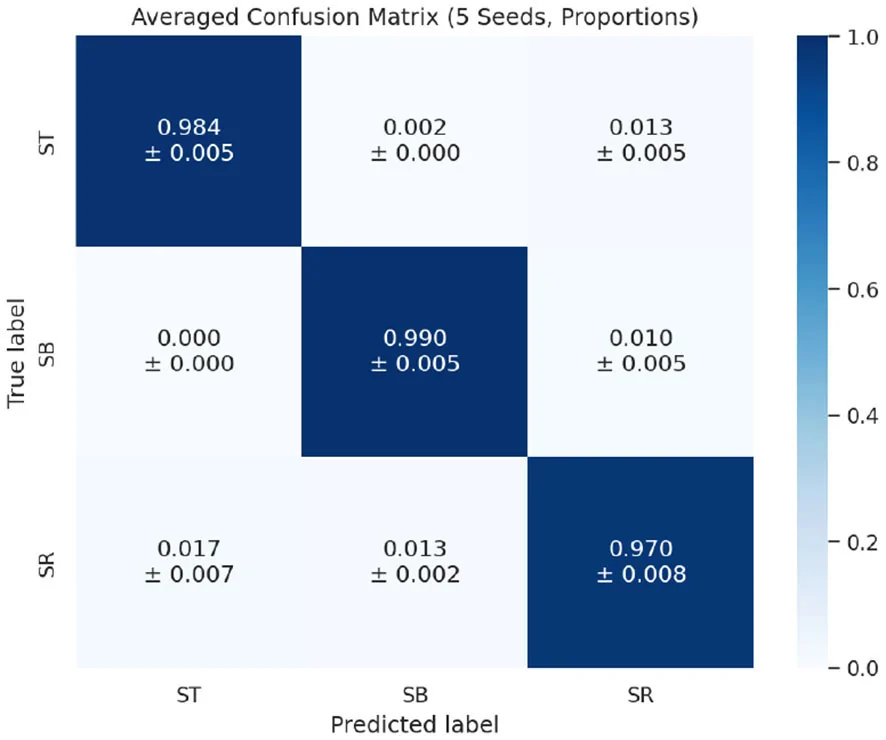
Normalized confusion matrix for the ResNet model on Data Type 1, showing class-wise prediction performance for ST, SB, and SR.

#### 3.3.3 Data Type 1 (non-pretrained)

When training the 2D ResNet architecture from scratch (without ImageNet pretraining) on Data Type 1, the model continues to exhibit exceptional learning capabilities on the raw ECG signals (Figure 13). Evaluated across five random seeds, the non-pretrained model attains a highly competitive average test accuracy of 98.23% ± 0.20% (Table 10). This indicates that while transfer learning provides a robust initialization, the network possesses sufficient capacity to learn discriminative temporal and spatial features directly from the OPI representations. Class-wise evaluation reveals strong predictive stability (Figure 14), with macro-averaged precision, recall, and F1-scores reaching 0.981, 0.983, and 0.982, respectively. Notably, the model achieves an outstanding F1-score of 0.9889 ± 0.0014 for the SB class, while maintaining robust performance on the more challenging ST (F1-score 0.9795 ± 0.0027) and SR (F1-score 0.9773 ± 0.0025) classes. Furthermore, the discriminative power remains near-optimal, as evidenced by ROC–AUC values of 0.9984 (ST), 0.9995 (SB), and 0.9985 (SR), resulting in a micro- and macro-averaged AUCs of 0.9990 and 0.9988, respectively. Overall, these findings demonstrate that the OPI-encoded Data Type 1 representations inherently provide a rich feature space, enabling the convolutional architecture to achieve highly competitive classification performance even in the absence of pretrained weights.

**Figure 13:**
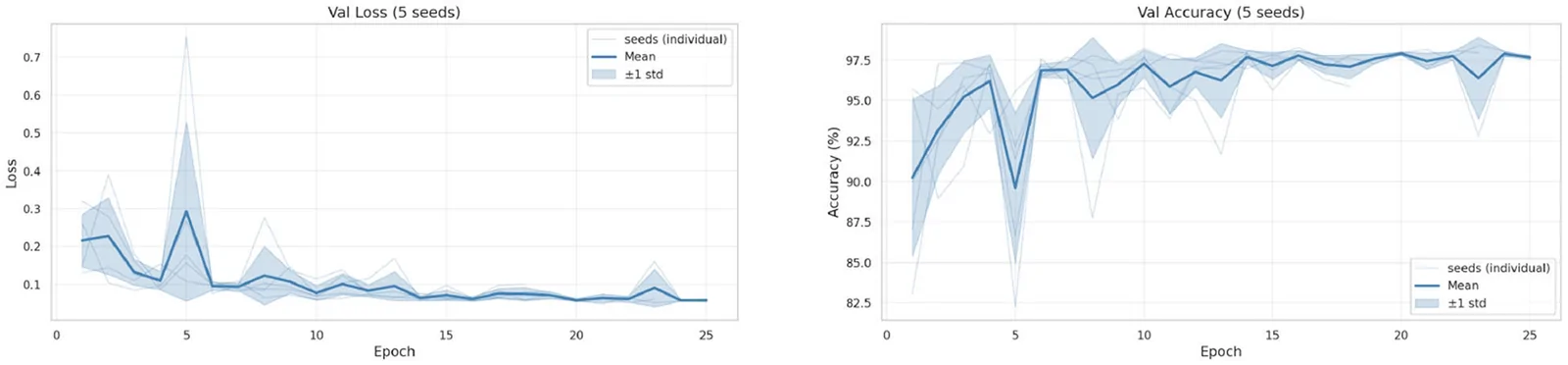
Validation losses **(Left)** and accuracies **(Right)** for the non-pretrained ResNet model on Data Type 1.

**Table 10:** Classification report for the non-pretrained ResNet model (Data Type 1).

Class	Precision	Recall	F1-score	Support
ST	0.9700 ± 0.0081	0.9893 ± 0.0043	0.9795 ± 0.0027	449
SB	0.9801 ± 0.0045	0.9979 ± 0.0025	0.9889 ± 0.0014	748
SR	0.9926 ± 0.0036	0.9626 ± 0.0066	0.9773 ± 0.0025	748
Accuracy			0.9823 ± 0.0020	1,945
Macro Avg	0.9809 ± 0.0023	0.9832 ± 0.0016	0.9819 ± 0.0021	1,945
Weighted Avg	0.9826 ± 0.0017	0.9823 ± 0.0020	0.9823 ± 0.0020	1,945

**Figure 14:**
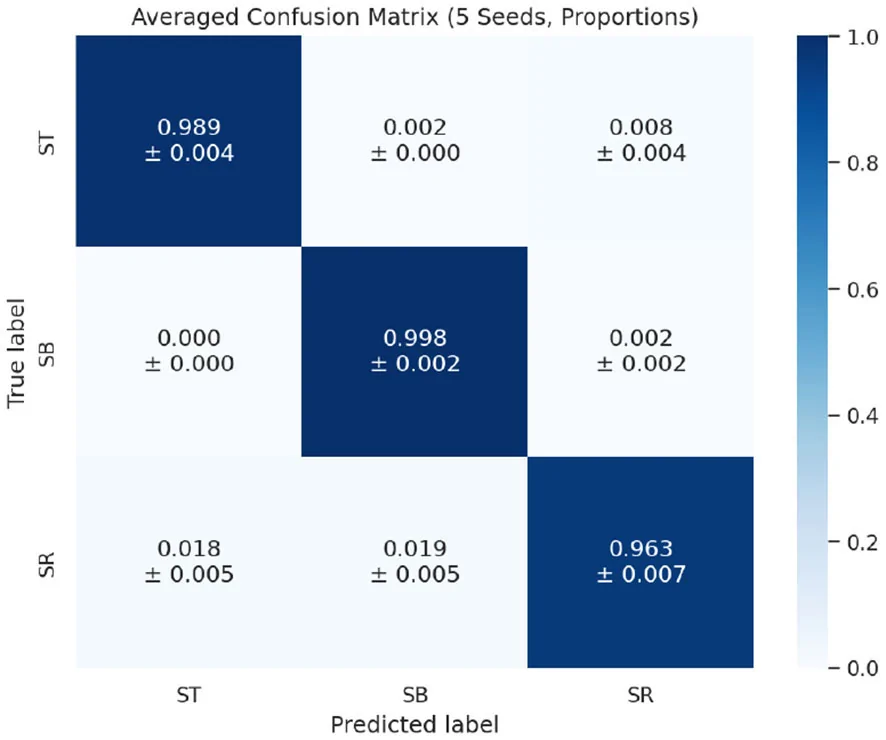
Normalized confusion matrix for the non-pretrained ResNet model on Data Type 1.

#### 3.3.4 Data Type 2

The 2D ResNet demonstrates strong and stable performance on the three-class ECG classification task, achieving rapid convergence during training (Figure 15). Evaluated across five random seeds, the model attains an average test accuracy of 97.50%±0.16%, confirming robust generalization performance, though marginally lower than that achieved by the Type 1 ResNet variant. Class-wise evaluation indicates consistently high predictive accuracy across all categories (Figure 16). The macro-averaged precision, recall, and F1-score are 0.975, 0.975, and 0.975, respectively, reflecting well-balanced classification, as summarized in Table 11. The ST and SB classes achieve particularly strong performance with F1-scores of 0.981 ± 0.003 and 0.980 ± 0.002, while the SR class remains comparatively more challenging, attaining an F1-score of 0.964 ± 0.002. Discriminative performance remains excellent, with ROC–AUC values of 0.9987 (ST), 0.9985 (SB), and 0.9954 (SR), yielding micro- and macro-averaged AUCs of *0.9977* and *0.9975*, respectively. Overall, these results confirm that the 2D ResNet architecture effectively captures discriminative temporal features in ECG signals.

**Figure 15:**
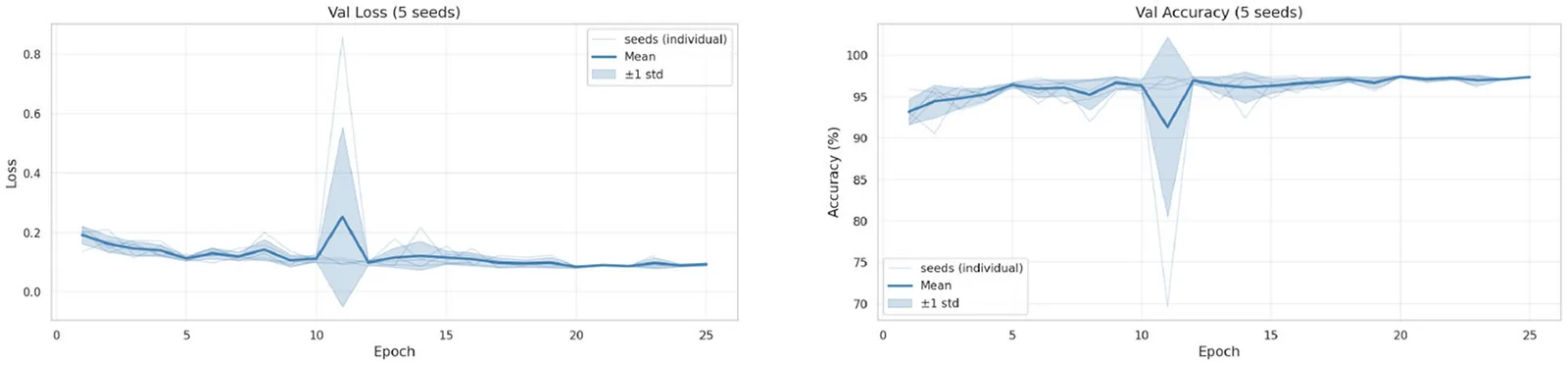
Validation losses **(Left)** and accuracies **(Right)** for the ResNet model on Data Type 2, illustrating stable convergence across epochs.

**Figure 16:**
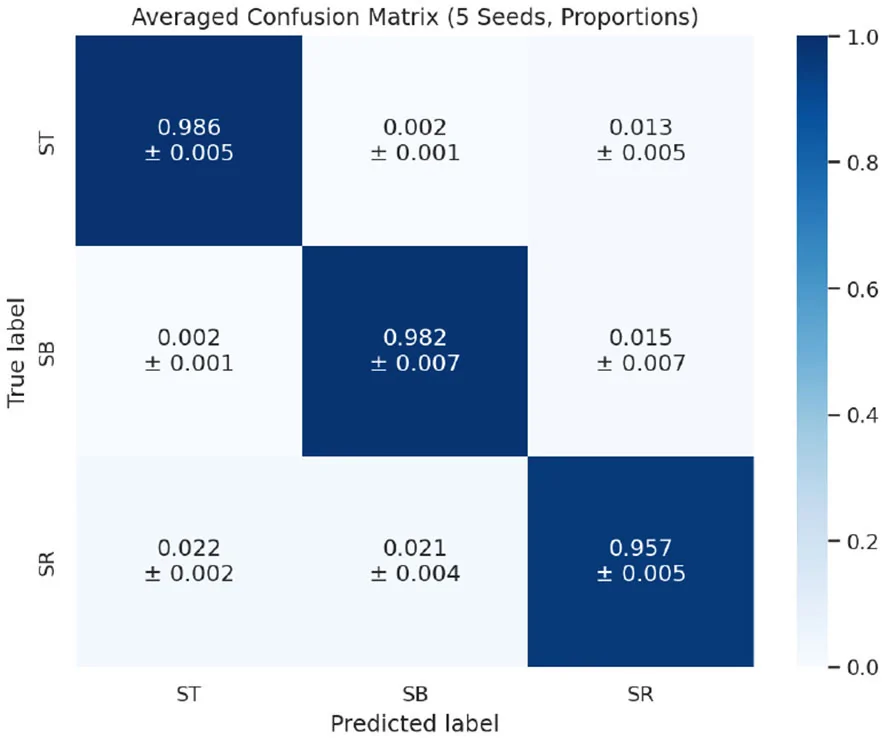
Normalized confusion matrix for the ResNet model on Data Type 2, showing class-wise prediction performance for ST, SB, and SR.

**Table 11:** Classification report for the ResNet model applied to 12-lead ECG signals (Data Type 2).

Class	Precision	Recall	F1-score	Support
ST	0.9759 ± 0.0021	0.9856 ± 0.0054	0.9807 ± 0.0028	748
SB	0.9778 ± 0.0046	0.9821 ± 0.0072	0.9799 ± 0.0015	750
SR	0.9713 ± 0.0062	0.9572 ± 0.0050	0.9642 ± 0.0022	748
Accuracy			0.9750 ± 0.0016	
Macro Avg	0.9750 ± 0.0016	0.9750 ± 0.0016	0.9749 ± 0.0016	2,246
Weighted Avg	0.9750 ± 0.0016	0.9750 ± 0.0016	0.9749 ± 0.0016	2,246

#### 3.3.5 Data Type 2 (non-pretrained)

For Data Type 2, the non-pretrained 2D ResNet achieves an average test accuracy of 97.39%±0.26% (Figure 17) across the five evaluation seeds (Table 12). Although marginally lower than the Type 1 configuration, this performance remains highly competitive with its pretrained counterpart, underscoring the architecture's ability to effectively extract relevant clinical patterns from the aggressively downsampled 50 × 50 OPI representations. The model exhibits well-balanced classification across all categories, yielding macro-averaged precision, recall, and F1-scores of 0.974. Analyzing the per-class metrics, the network correctly identifies the ST and SB classes with high reliability (Figure 18), achieving F1-scores of 0.9812 ± 0.0015 and 0.9793 ± 0.0035, respectively. The SR class, inherently presenting more complex, comorbid representations, achieves an F1-score of 0.9612 ± 0.0028. The receiver operating characteristic analysis confirms strong separability, with AUC values reaching 0.9983 for ST, 0.9984 for SB, and 0.9944 for SR, culminating in a micro- and macro-averaged AUCs of 0.9973 and 0.9970, respectively. Consequently, the results indicate that even without the inductive bias provided by transfer learning, the ResNet model successfully captures the critical morpho-temporal variations present in Data Type 2.

**Figure 17:**
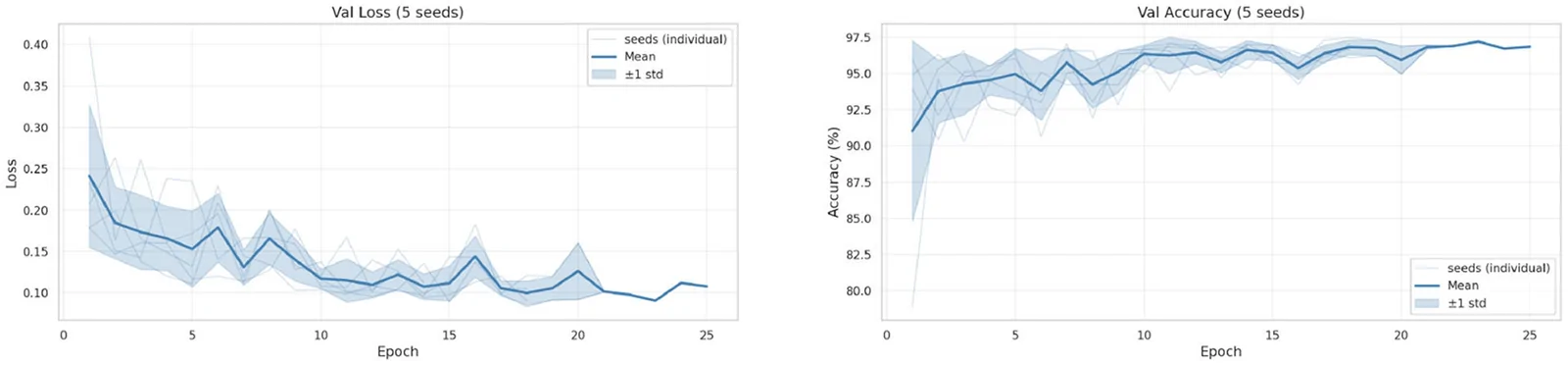
Validation losses **(Left)** and accuracies **(Right)** for the non-pretrained ResNet model on Data Type 2.

**Table 12:** Classification report for the non-pretrained ResNet model (Data Type 2).

Class	Precision	Recall	F1-score	Support
ST	0.9800 ± 0.0048	0.9824 ± 0.0063	0.9812 ± 0.0015	748
SB	0.9788 ± 0.0074	0.9800 ± 0.0115	0.9793 ± 0.0035	750
SR	0.9633 ± 0.0133	0.9594 ± 0.0084	0.9612 ± 0.0028	748
Accuracy			0.9739 ± 0.0026	2,246
Macro Avg	0.9740 ± 0.0025	0.9739 ± 0.0025	0.9739 ± 0.0025	2,246
Weighted Avg	0.9740 ± 0.0026	0.9739 ± 0.0026	0.9739 ± 0.0026	2,246

**Figure 18:**
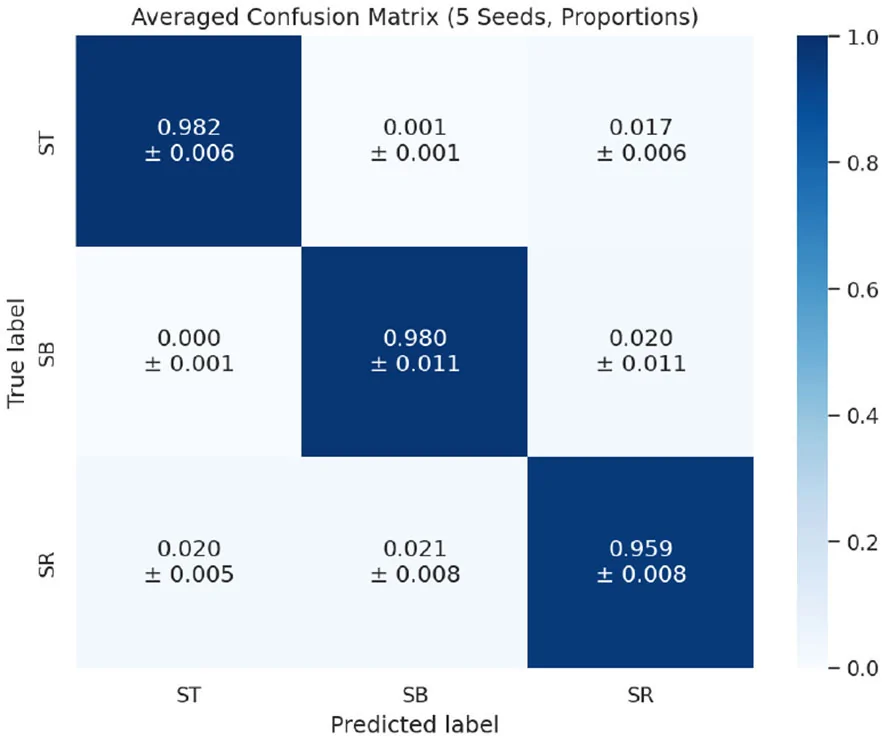
Normalized confusion matrix for the non-pretrained ResNet model on Data Type 2.

### 3.4 Baseline 2 : 1D transformer

We employ a vanilla Transformer architecture (Section 2.2.3) denoted as VanillaTransformerECG as the second baseline to evaluate OPI representations against attention-based sequence modeling. While ResNet-18 captures local temporal patterns through convolutional operations, the Transformer leverages self-attention to model long-range dependencies across the entire ECG signal. The raw 12-lead ECG input of shape (12, 5,000) is tokenized into patches and processed through multi-head self-attention layers, enabling the model to capture global temporal correlations and inter-lead relationships. This baseline complements the convolutional approach by representing a different architectural paradigm that has demonstrated strong performance on sequential data. Comparing OPI representations against both convolutional (ResNet-18) and attention-based (Transformer) baselines provides a comprehensive evaluation across different inductive biases—local feature extraction vs. global context modeling—ensuring that observed performance differences arise from the representation itself rather than architectural limitations.

#### 3.4.1 Normalization

We apply the same normalization procedure as described in the Normalization paragraph of Section 2.2.4 (ResNet baseline) for the Transformer baseline, ensuring consistent preprocessing across all models to facilitate fair comparisons.

#### 3.4.2 Data Type 1

The vanilla Transformer-based ECG classifier with 12 leads and a patch-based attention mechanism achieved strong and stable performance on the three-class classification task (ST, SB, SR) (Figure 19). Evaluated across five random seeds, the model achieved an average test accuracy of 93.00% ± 0.75% (Table 13), demonstrating that the learned representations transfer well beyond the validation set despite mild variance. Detailed evaluation metrics further confirm the robustness of the model (Figure 20). The macro-averaged precision, recall, and F1-score were 0.928, 0.934, and 0.931, respectively, with balanced performance across classes. Class-wise ROC-AUC scores were consistently high (ST: 0.9934 ± 0.0004, SB: 0.9917 ± 0.0010, SR: 0.9779 ± 0.0027), yielding a macro-average AUC of 0.9877 and a micro-average AUC of 0.9892 ± 0.0012. The micro-averaged precision–recall curve achieved an average precision of 0.9793 ± 0.0026, indicating excellent discrimination even under class imbalance. Overall, these results show that a relatively lightweight vanilla Transformer can effectively capture clinically relevant temporal patterns in long ECG signals.

**Figure 19:**
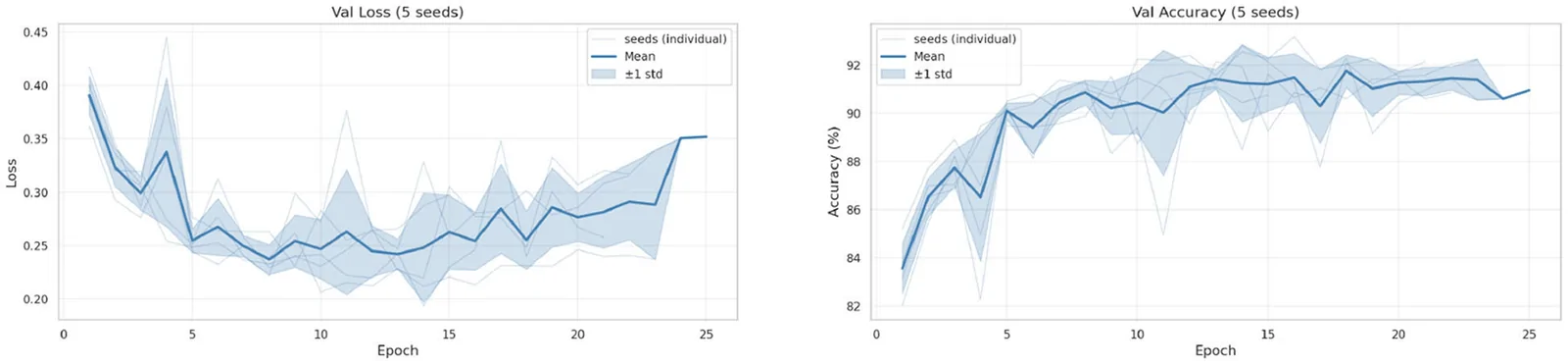
Validation losses **(Left)** and accuracies **(Right)** for the Transformer model on Data Type 1.

**Table 13:** Classification report for the Transformer model applied to 12-lead ECG signals (Data Type 1).

Class	Precision	Recall	F1-score	Support
ST (Sinus Tachycardia)	0.9164 ± 0.0118	0.9635 ± 0.0110	0.9392 ± 0.0054	449
SB (Sinus Bradycardia)	0.9416 ± 0.0103	0.9492 ± 0.0168	0.9452 ± 0.0063	748
SR (Sinus Rhythm)	0.9274 ± 0.0163	0.8906 ± 0.0136	0.9085 ± 0.0087	748
Accuracy			0.9300 ± 0.0075	
Macro Avg	0.9285 ± 0.0072	0.9344 ± 0.0057	0.9310 ± 0.0072	1,945
Weighted Avg	0.9303 ± 0.0075	0.9300 ± 0.0075	0.9297 ± 0.0075	1,945

**Figure 20:**
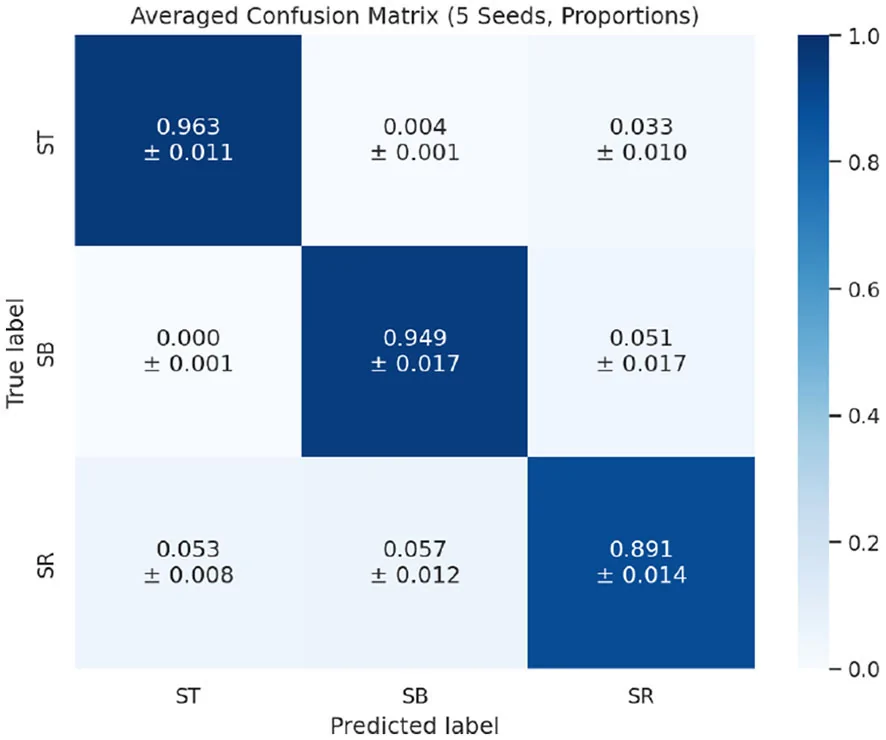
Normalized confusion matrix for the Transformer model on Data Type 1, showing class-wise prediction performance for ST, SB, and SR.

#### 3.4.3 Data Type 2

The vanilla Transformer exhibits stable and reliable performance on the three-class ECG classification task (Figure 21). Evaluated across five random seeds, the model yields an average test accuracy of 91.33%±1.07% (Table 14), which indicates good generalization, albeit slightly lower than that observed for the Type 1 Transformer configuration. Quantitative evaluation reveals balanced performance across classes (Figure 22), with macro-averaged precision, recall, and F1-score all averaging 0.915, 0.913, and 0.914, respectively. The ST and SB classes achieve strong classification performance (F1-scores of 0.938 ± 0.006 and 0.928 ± 0.010, respectively), whereas the SR class remains comparatively more challenging, with an F1-score of 0.875 ± 0.012. Receiver operating characteristic analysis shows consistently high discriminative power, with class-wise AUC values of 0.9906 ± 0.0009 (ST), 0.9904 ± 0.0017 (SB), and 0.9699 ± 0.0041 (SR), resulting in a macro-average AUC of 0.9836 and a micro-average AP of 0.9689 ± 0.0053. Overall, these results demonstrate that the vanilla Transformer effectively captures long-range temporal dependencies in ECG signals, while highlighting residual class-specific performance disparities.

**Figure 21:**
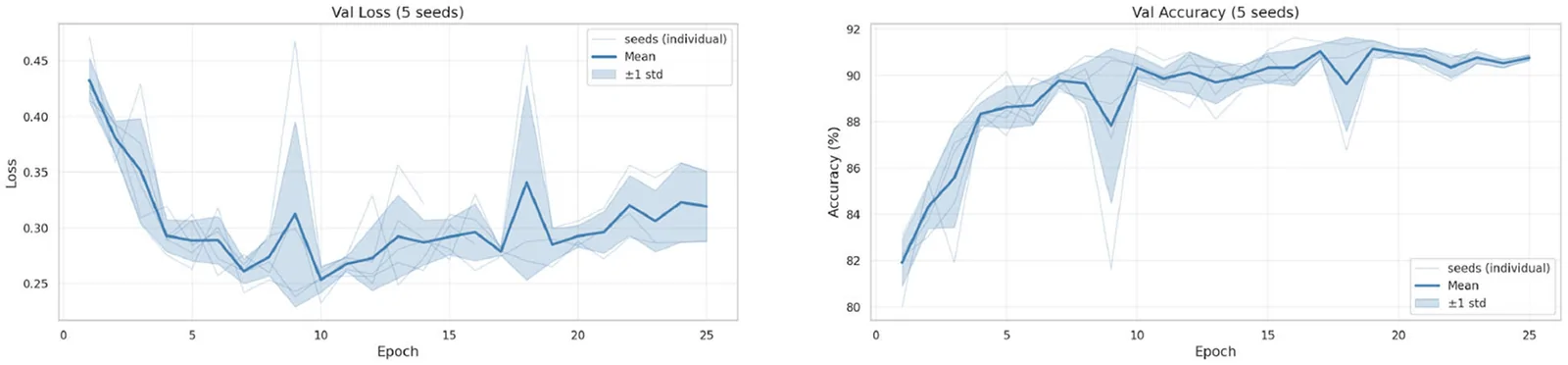
Validation losses **(Left)** and accuracies **(Right)** for the Transformer model on Data Type 2.

**Table 14:** Classification report for the Transformer model applied to 12-lead ECG signals (Data Type 2).

Class	Precision	Recall	F1-score	Support
ST	0.9406 ± 0.0079	0.9361 ± 0.0195	0.9381 ± 0.0062	748
SB	0.9425 ± 0.0093	0.9131 ± 0.0145	0.9275 ± 0.0104	750
SR	0.8606 ± 0.0245	0.8906 ± 0.0106	0.8751 ± 0.0123	748
Accuracy			0.9133 ± 0.0096	2,246
Macro Avg	0.9146 ± 0.0084	0.9133 ± 0.0096	0.9136 ± 0.0093	2,246
Weighted Avg	0.9146 ± 0.0084	0.9133 ± 0.0096	0.9136 ± 0.0093	2,246

**Figure 22:**
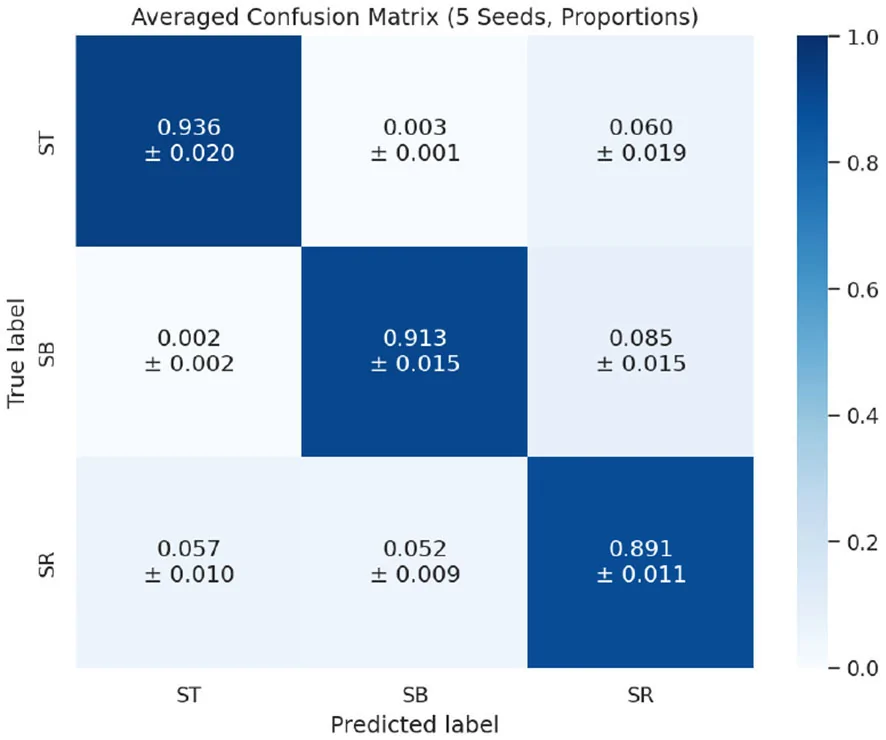
Normalized confusion matrix for the Transformer model on Data Type 2, showing class-wise prediction performance for ST, SB, and SR.

### 3.5 Baseline 3 : single-lead ECG classification using FFNN

We next report results on single-lead ECG classification using a feed-forward neural network (FFNN) described in Subsection 2.2.1. This analysis focuses on Lead 1 signals and evaluates model performance without any polynomial encoding. We present results separately for Data Type 1 and Data Type 2, illustrating the impact of signal variability and comorbidities on classification accuracy.

#### 3.5.1 Data Type 1: lead 1 signals

To evaluate the performance of a feed-forward neural network (FFNN) on single-lead ECG data, we trained the model using raw Lead 1 signals from Data Type 1. The dataset consisted of *2991* samples for ST, *4986* samples for SB, and *4988* samples for SR, split into 70–15–15 for training-validation-test.

The FFNN trained on single-lead Data Type 1 exhibited gradual convergence over the full 25-epoch schedule (Figure 23). Evaluated across five random seeds, the model achieved an average test accuracy of 82.30%±0.78%, indicating limited generalization capacity for single-lead inputs compared to multi-lead representations. As shown in Figure 24, the model achieved recall values of 70.24%±3.01% for ST, 90.35%±0.83% for SB, and 81.50%±3.04% for SR, with SB emerging as the most separable class. Table 15 reports an overall accuracy of 82.3%, with macro- and weighted-average F1-scores of 0.806 and 0.824, respectively, substantially lower than multi-lead or encoded representations. ROC analysis (Supplementary Figure 11a) yielded AUC values of 0.9025 ± 0.0058 (ST), 0.9699 ± 0.0026 (SB), and 0.9317 ± 0.0048 (SR), with a micro-averaged AUC of 0.9421 ± 0.0035 and a macro-averaged AUC of 0.9347. The micro-averaged precision–recall score of 0.9011 ± 0.0068 further reflects the reduced discriminative power of single-lead FFNN models.

**Figure 23:**
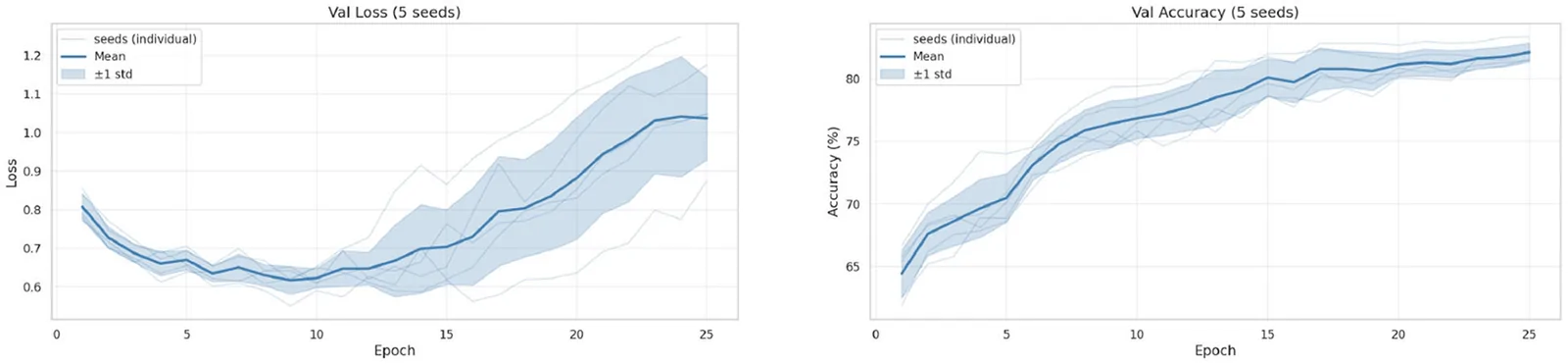
Validation losses **(Left)** and accuracies **(Right)** for the single-lead FFNN on Lead 1 signals (Data Type 1).

**Figure 24:**
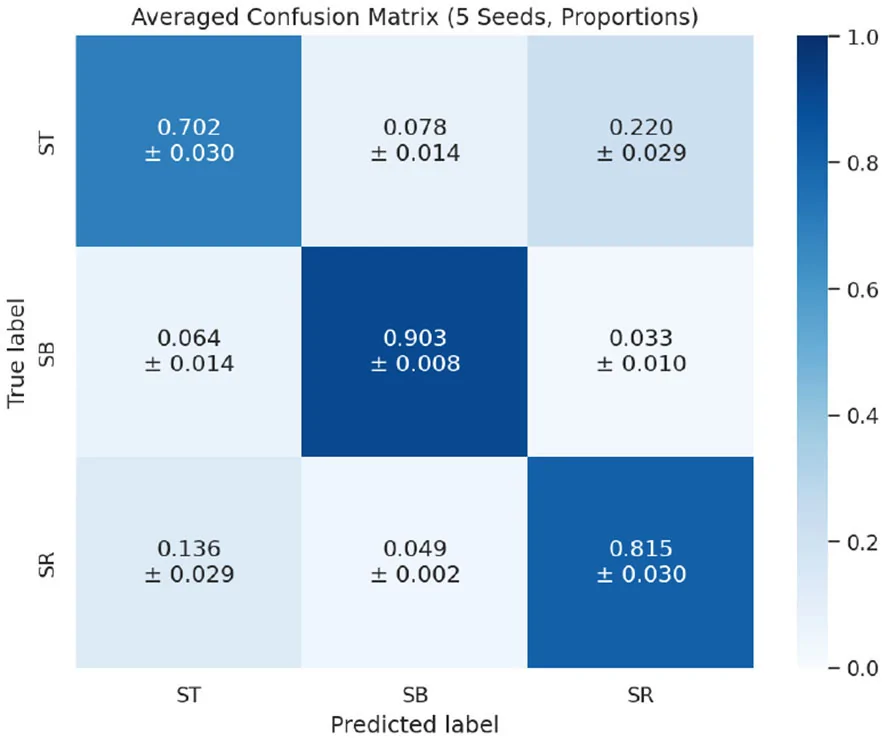
Confusion matrix for single-lead FFNN classification on Data Type 1.

**Table 15:** Classification report for the single-lead FFNN on Data Type 1.

Class	Precision	Recall	F1-score	Support
ST	0.6814 ± 0.0334	0.7024 ± 0.0301	0.6904 ± 0.0078	449
SB	0.9043 ± 0.0066	0.9035 ± 0.0083	0.9038 ± 0.0037	748
SR	0.8332 ± 0.0197	0.8150 ± 0.0304	0.8232 ± 0.0086	748
Accuracy			0.8230 ± 0.0078	1,945
Macro Avg	0.8063 ± 0.0058	0.8070 ± 0.0027	0.8058 ± 0.0061	1,945
Weighted Avg	0.8255 ± 0.0019	0.8230 ± 0.0078	0.8236 ± 0.0054	1,945

#### 3.5.2 Data Type 2: lead 1 signals

For Data Type 2, which includes additional comorbidities, the FFNN was trained on the same Lead 1 signals. The dataset consisted of *4987* ST, *4996* SB, and *4984* SR samples, with a 70–15–15 train-validation-test split.

The FFNN trained on single-lead Data Type 2 exhibited steady convergence, with training accuracy continuing to improve (Figure 25) while validation performance saturated, indicating mild overfitting. Evaluated across five random seeds, the model achieved an average test accuracy of 77.52%±0.39%. The confusion matrix in Figure 26 shows moderate class-wise performance, with recall values of 73.82%±2.54% for ST, 78.96%±0.76% for SB, and 79.76%±2.76% for SR. As summarized in Table 16, the model achieved an overall accuracy of 77.5%, with macro- and weighted-average F1-scores of approximately 0.776. The ROC curves in Supplementary Figure 12a indicate reasonable discriminative capability, with AUC values of 0.8865 ± 0.0046 (ST), 0.9309 ± 0.0031 (SB), and 0.9163 ± 0.0052 (SR), and micro- and macro-averaged AUCs of *0.9115* and *0.9112*, respectively. The micro-averaged precision–recall curve in Supplementary Figure 12b yields an average precision of 0.8545 ± 0.0061, reflecting the limitations of single-lead inputs under increased signal variability.

**Figure 25:**
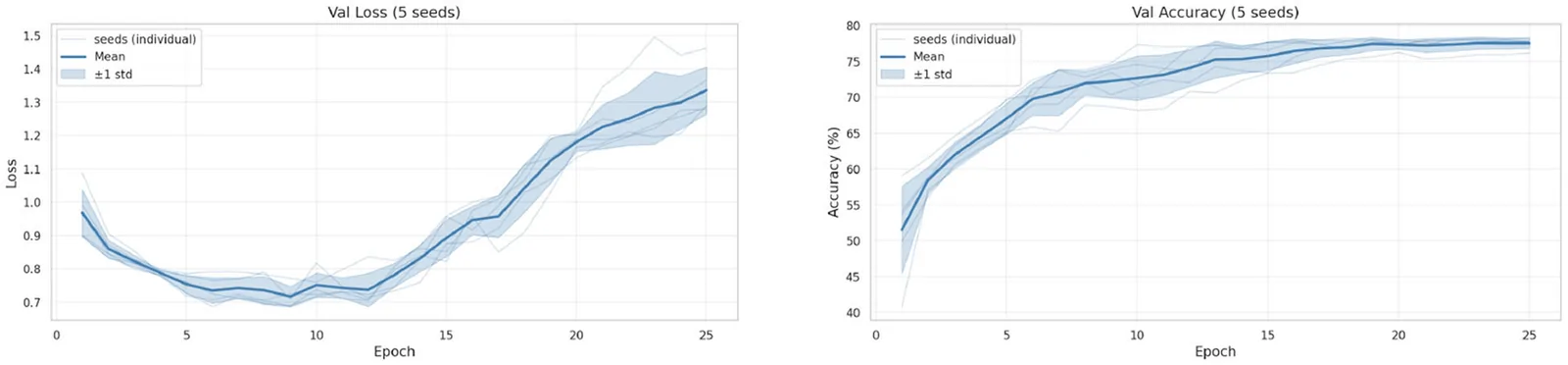
Validation losses **(Left)** and accuracies **(Right)** for the FFNN on single-lead Data Type 2 over 25 epochs.

**Figure 26:**
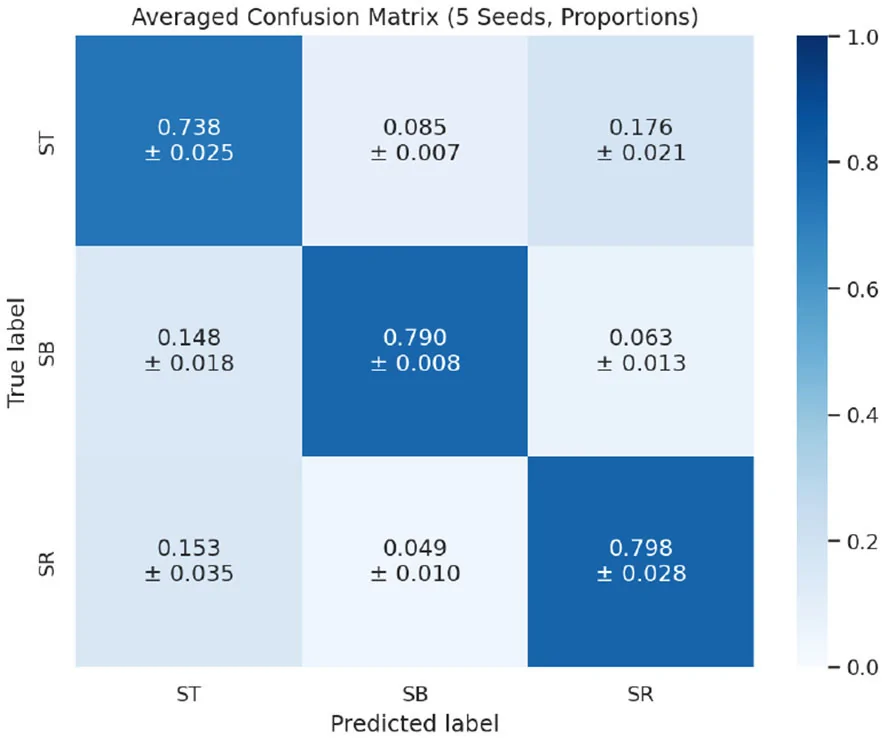
Confusion matrix for FFNN classification on single-lead Data Type 2.

**Table 16:** Classification report for the FFNN model on single-lead Data Type 2.

Class	Precision	Recall	F1-score	Support
ST	0.7118 ± 0.0272	0.7382 ± 0.0254	0.7239 ± 0.0079	748
SB	0.8553 ± 0.0151	0.7896 ± 0.0076	0.8211 ± 0.0076	750
SR	0.7702 ± 0.0180	0.7976 ± 0.0276	0.7830 ± 0.0066	748
Accuracy			0.7752 ± 0.0035	2,246
Macro Avg	0.7791 ± 0.0029	0.7751 ± 0.0035	0.7760 ± 0.0030	2,246
Weighted Avg	0.7792 ± 0.0029	0.7752 ± 0.0035	0.7760 ± 0.0030	2,246

### 3.6 Baseline 4 : 12-lead raw ECG classification using 1D-ResNet

To establish a strong baseline that operates directly on the temporal ECG signal, we employed the ResNet1D implementation available in the SelfEEG library (Del Pup et al., 2024), which we denote as SelfEEGResNet1D. This architecture is a residual one-dimensional convolutional neural network specifically designed for time-series analysis and therefore processes the ECG waveform without relying on any spatial encoding or transformation. The model receives the full 12-lead ECG recording as input with dimensionality 12 × 5,000, and all convolutional operations are applied along the temporal axis to capture multi-scale temporal patterns present in the signal. Additional architectural details of the network are provided in Section 2.2.5.

#### 3.6.1 Data Type 1

The 1D-ResNet architecture with 12 ECG leads demonstrates strong and stable performance on the three-class rhythm classification task (ST, SB, SR). During training, the model converges rapidly (Figure 27), with validation accuracy improving from approximately 93.11% in the first epoch to a peak of 98.56%. While the training accuracy continues to increase up to approximately 98.88%, the validation accuracy stabilizes near 98%, indicating only minimal overfitting. Evaluated across five random seeds, the model achieves an average test accuracy of 98.40% ± 0.28% (Table 17), demonstrating excellent generalization capability. The macro-averaged precision, recall, and F1-score are 0.9826, 0.9841, and 0.9833, respectively, indicating highly balanced classification performance (Figure 28). Class-wise ROC-AUC values are extremely high (ST: 0.9984 ± 0.0002, SB: 0.9995 ± 0.0001, SR: 0.9989 ± 0.0003), yielding micro- and macro-averaged AUC scores of *0.9991* and *0.9989*, respectively. The micro-averaged precision–recall curve achieves an average precision of 0.9985 ± 0.0002, confirming the model's strong discriminative capability even under potential class imbalance.

**Figure 27:**
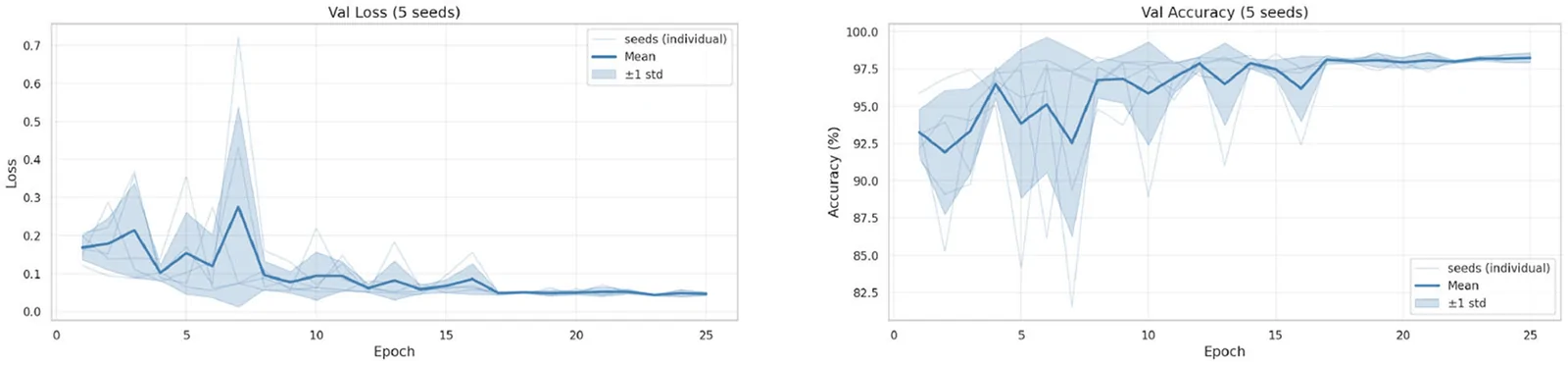
Validation losses **(Left)** and accuracies **(Right)** for the 1D-ResNet (Data Type 1).

**Table 17:** Classification report for the 1D-ResNet Data Type 1.

Class	Precision	Recall	F1-score	Support
ST	0.9732 ± 0.0075	0.9853 ± 0.0044	0.9792 ± 0.0024	449
SB	0.9848 ± 0.0072	0.9976 ± 0.0016	0.9911 ± 0.0029	748
SR	0.9899 ± 0.0035	0.9695 ± 0.0094	0.9796 ± 0.0034	748
Accuracy			0.9840 ± 0.0028	
Macro Avg	0.9826 ± 0.0027	0.9841 ± 0.0019	0.9833 ± 0.0026	1,945
Weighted Avg	0.9841 ± 0.0024	0.9840 ± 0.0028	0.9839 ± 0.0028	1,945

**Figure 28:**
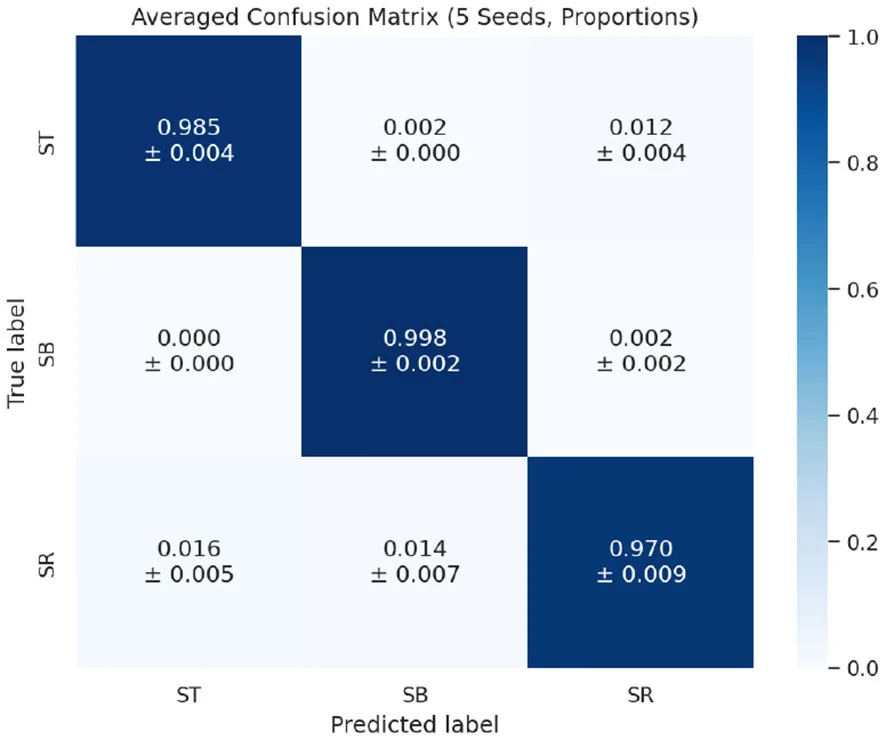
Confusion matrix for 1D-ResNet Data Type 1.

#### 3.6.2 Data Type 2

The 1D-ResNet architecture evaluated on the Type 2 ECG dataset also demonstrates strong performance on the three-class rhythm classification task (ST, SB, SR). During training, the model converges steadily (Figure 29), with validation accuracy increasing from approximately 95.10% in the initial epoch to a peak of 98.08%. While the training accuracy continues to increase up to approximately 98.18%, the validation accuracy stabilizes close to 98%, indicating good generalization with minimal overfitting. Evaluated across five random seeds, the model achieves an average test accuracy of 97.78%±0.20% (Table 18). The macro-averaged precision, recall, and F1-score are all averaging 0.9779, 0.9778, and 0.9778, respectively, demonstrating balanced classification performance across classes (Figure 30). The class-wise F1-scores are 0.9806 ± 0.0019 for ST, 0.9857 ± 0.0018 for SB, and 0.9671 ± 0.0024 for SR, indicating slightly lower performance for the SR class but still strong overall accuracy. Receiver operating characteristic analysis shows consistently high discriminative capability, with AUC values of 0.9986 ± 0.0002 (ST), 0.9994 ± 0.0001 (SB), and 0.9970 ± 0.0003 (SR). These results yield a micro- and macro-averaged AUCs of *0.9984* and *0.9983*, respectively. The micro-averaged precision–recall curve achieves an average precision of 0.9969 ± 0.0004, confirming the model's strong ability to distinguish between rhythm classes even under potential class imbalance.

**Figure 29:**
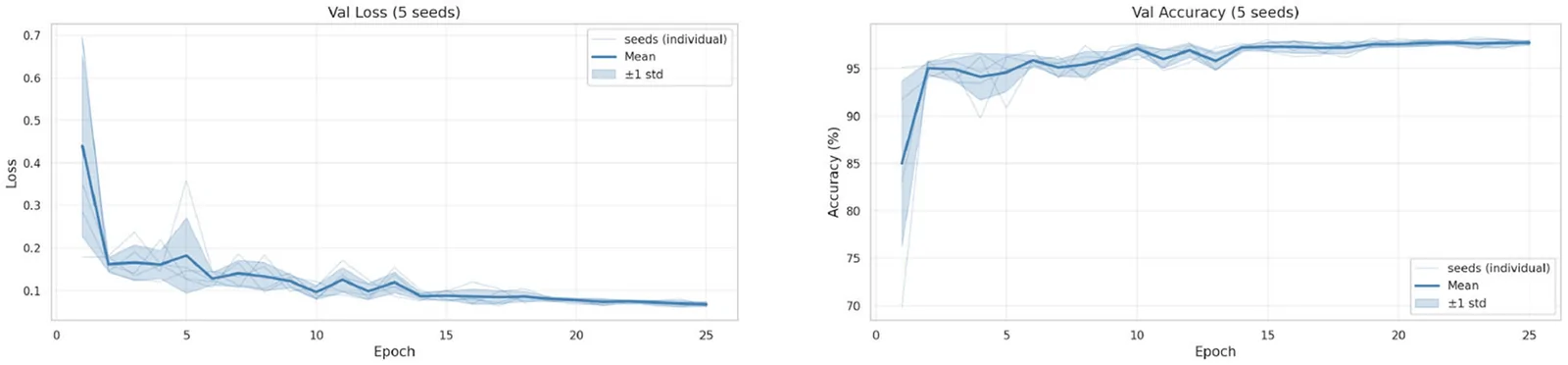
Validation losses **(Left)** and accuracies **(Right)** for the 1D-ResNet (Data Type 2).

**Table 18:** Classification report for the 1D-ResNet Data Type 2.

Class	Precision	Recall	F1-score	Support
ST	0.9797 ± 0.0030	0.9816 ± 0.0029	0.9806 ± 0.0019	748
SB	0.9874 ± 0.0024	0.9840 ± 0.0048	0.9857 ± 0.0018	750
SR	0.9664 ± 0.0051	0.9679 ± 0.0024	0.9671 ± 0.0024	748
Accuracy			0.9778 ± 0.0018	2,246
Macro Avg	0.9779 ± 0.0018	0.9778 ± 0.0018	0.9778 ± 0.0018	2,246
Weighted Avg	0.9779 ± 0.0018	0.9778 ± 0.0018	0.9778 ± 0.0018	2,246

**Figure 30:**
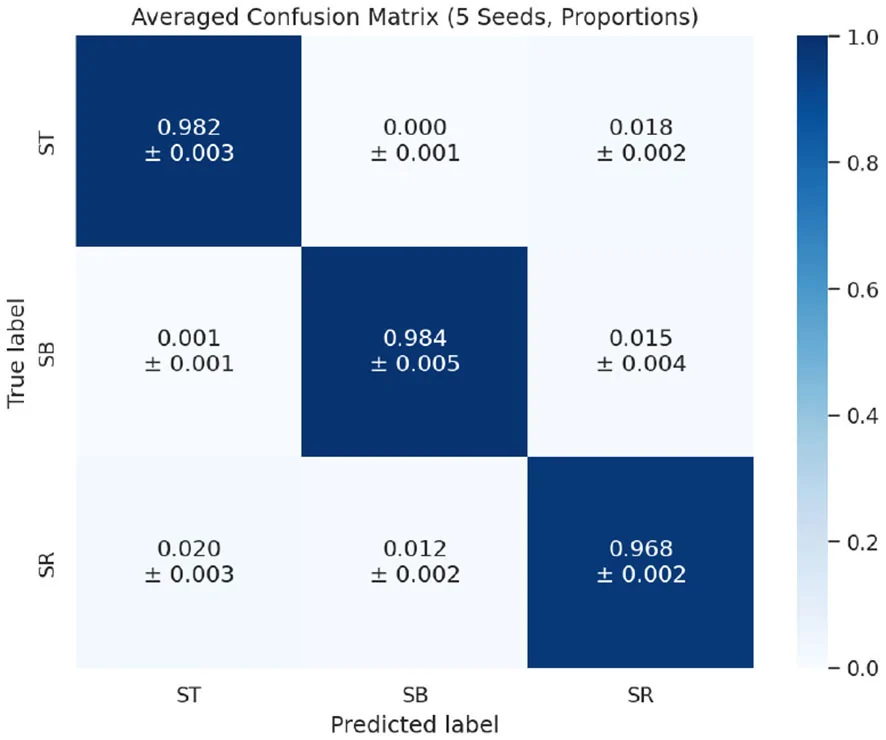
Confusion matrix for 1D Resnet Data Type 2.

### 3.7 Baseline 5: single-lead raw ECG classification using 1D-ResNet

To evaluate the performance of the ResNet1D model on single-lead data, we trained and tested the architecture using only the first lead (Lead 1) of the ECG signals. This baseline assesses the capability of the 1D-ResNet to extract discriminative temporal features without the spatial context provided by the full 12-lead setup.

#### 3.7.1 Data Type 1

Evaluated across five random seeds, the 1D-ResNet trained on single-lead Data Type 1 achieved an average test accuracy of 98.03%±0.24% (Table 19). The macro-averaged precision, recall, and F1-score were 0.9782, 0.9819, and 0.9799, respectively. Class-wise F1-scores were high across all categories (Figure 31): 0.9774 ± 0.0026 for ST, 0.9874 ± 0.0025 for SB, and 0.9748 ± 0.0027 for SR. The model exhibited strong discriminative capability, with a macro-average AUC of 0.9981 and a micro-averaged precision-recall score of 0.9972 ± 0.0011. These results indicate that even with a single lead, the 1D-ResNet effectively captures the essential temporal patterns for this classification task. A seed-level paired *t*-test comparing the 12-lead 1D-ResNet (98.40%±0.28%) against the single-lead 1D-ResNet (98.03%±0.24%) on Data Type 1 yields a mean difference of +0.37% (*t*(4) = 1.629, *p* = 0.179), confirming that the performance gap between multi-lead and single-lead processing on Data Type 1 is not statistically significant.

**Table 19:** Classification report for single-lead 1D-ResNet on Data Type 1.

Class	Precision	Recall	F1-score	Support
ST	0.9629 ± 0.0051	0.9924 ± 0.0023	0.9774 ± 0.0026	449
SB	0.9876 ± 0.0080	0.9874 ± 0.0108	0.9874 ± 0.0025	748
SR	0.9841 ± 0.0114	0.9658 ± 0.0084	0.9748 ± 0.0027	748
Accuracy			0.9803 ± 0.0024	1,945
Macro Avg	0.9782 ± 0.0022	0.9819 ± 0.0021	0.9799 ± 0.0023	1,945
Weighted Avg	0.9806 ± 0.0022	0.9803 ± 0.0024	0.9802 ± 0.0024	1,945

**Figure 31:**
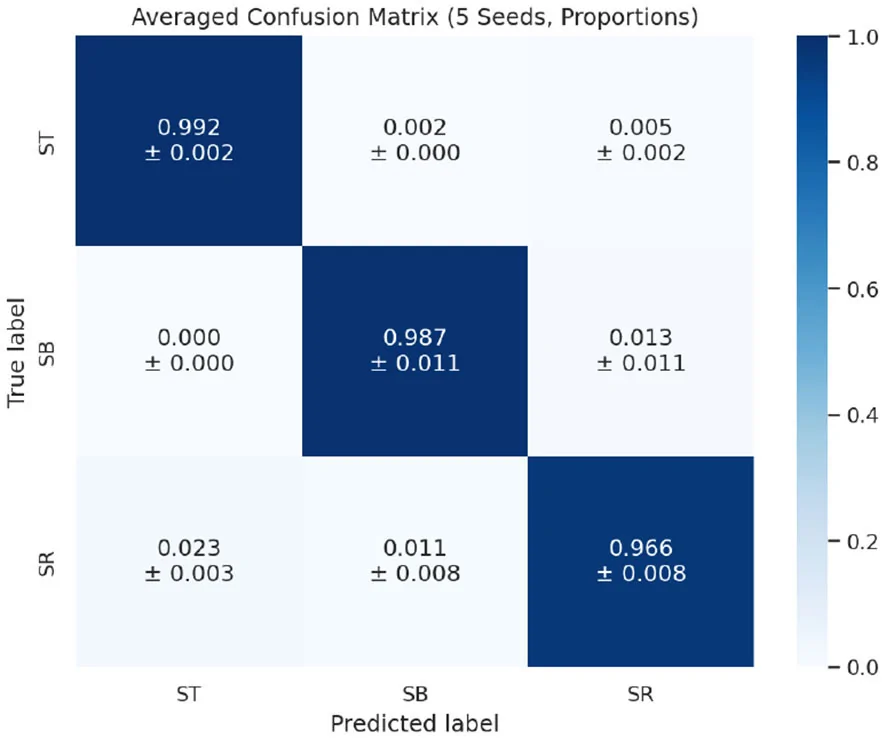
Confusion matrix for single-lead 1D-ResNet on Data Type 1.

#### 3.7.2 Data Type 2

For Data Type 2, the single-lead 1D-ResNet maintained robust performance, achieving an average test accuracy of 97.28%±0.12% across the five evaluation seeds (Table 20). The macro-averaged precision, recall, and F1-score were 0.9728, 0.9727, and 0.9727 respectively. Class-wise analysis revealed F1-scores of (Figure 32) 0.9791 ± 0.0014 for ST, 0.9780 ± 0.0012 for SB, and 0.9610 ± 0.0016 for SR. The model achieved a macro-average AUC of 0.9969 and a micro-average precision-recall score of 0.9948 ± 0.0007, demonstrating its resilience to the added complexities in Data Type 2 when processing single-lead inputs. In contrast to Data Type 1, a seed-level paired *t*-test comparing the 12-lead 1D-ResNet (97.78%±0.20%) against the single-lead 1D-ResNet (97.28%±0.12%) on Data Type 2 yields a mean difference of +0.51% (*t*(4) = 6.626, *p* = 0.0027), confirming that multi-lead integration provides a statistically significant improvement (*p* < 0.01) when diagnosing comorbidity cases.

**Table 20:** Classification report for single-lead 1D-ResNet on Data Type 2.

Class	Precision	Recall	F1-score	Support
ST	0.9756 ± 0.0053	0.9826 ± 0.0050	0.9791 ± 0.0014	748
SB	0.9727 ± 0.0063	0.9835 ± 0.0071	0.9780 ± 0.0012	750
SR	0.9701 ± 0.0049	0.9521 ± 0.0055	0.9610 ± 0.0016	748
Accuracy			0.9728 ± 0.0011	2,246
Macro Avg	0.9728 ± 0.0011	0.9727 ± 0.0011	0.9727 ± 0.0011	2,246
Weighted Avg	0.9728 ± 0.0011	0.9728 ± 0.0011	0.9727 ± 0.0011	2,246

**Figure 32:**
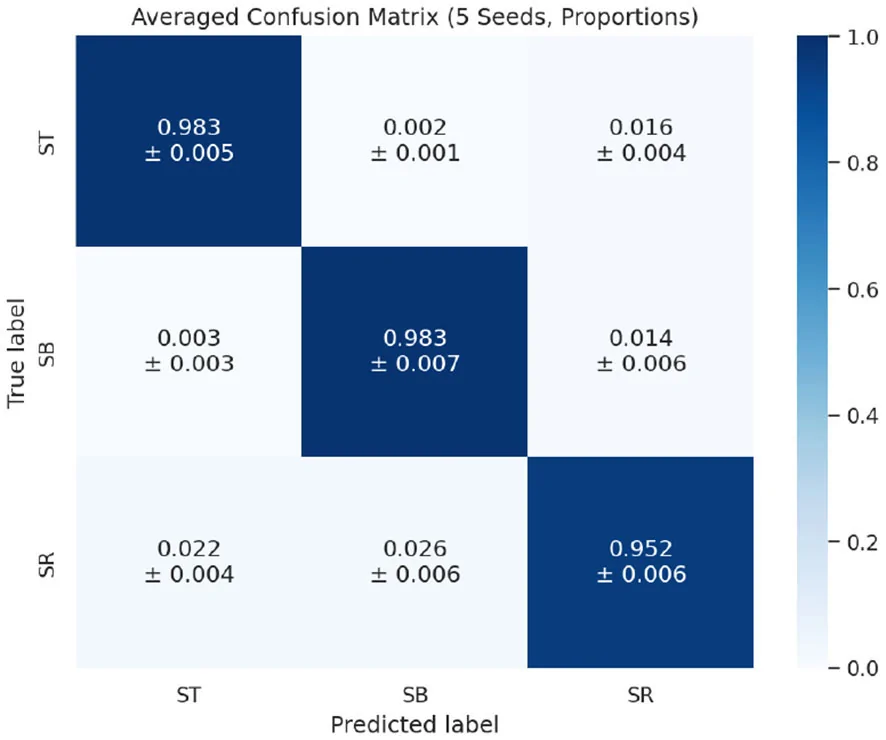
Confusion matrix for single-lead 1D-ResNet on Data Type 2.

### 3.8 Alternative orthogonal polynomial encodings

In addition to the Legendre-based superposition encoding, we systematically explored other families of orthogonal polynomials to investigate whether the choice of basis functions impacts model performance. Specifically, we evaluated *Chebyshev* encoding, keeping the downstream CNN architecture fixed for fair comparison.

#### 3.8.1 Model performance on Type 1

To construct compact representations of ECG reports, we employed Chebyshev polynomials of the first kind, as orthogonal basis functions on [−1, 1]. Each of the 12 leads was projected onto the Chebyshev basis, and the resulting signals were superposed to form a 5,000 × 5,000 image, subsequently downsampled to 100 × 100 using coarse-graining. The CNN architecture was identical to that used in the Legendre-based experiments and was trained using CrossEntropyLoss with the Adam optimizer (learning rate η = 1 × 10^−3^, weight decay = 10^−4^). A learning rate scheduler (ReduceLROnPlateau, factor = 0.5, patience = 2) and early stopping (patience = 7) were employed, with training capped at 25 epochs. The best-performing model, selected based on validation accuracy, was retained for evaluation.

For Data Type 1, the CNN trained on Chebyshev-encoded images exhibited stable convergence (Figure 33). Evaluated across five random seeds, the model achieved an average test accuracy of 98.34%±0.13%. The confusion matrix (Figure 34) shows strong class-wise performance, with recall values of *98.98%* ± *0.41%* for ST, *99.87%* ± *0.15%* for SB, and *96.44%* ± *0.23%* for SR. As summarized in Table 21, the model attained an overall accuracy of *98.34%* ± *0.13%*, with macro- and weighted-average F1-scores of approximately *0.983*, indicating balanced performance across classes. The ROC analysis (Supplementary Figure 15a) confirms excellent discriminative capability, with class-wise AUCs of *0.9987* ± *0.0001* (ST), *0.9997* ± *0.0001* (SB), and *0.9989* ± *0.0003* (SR), and micro- and macro-averaged AUCs of *0.9992* and *0.9991*, respectively. The micro-averaged average precision score also reached *0.9986* ± *0.0002* (Supplementary Figure 15b), underscoring the robustness of the model.

**Figure 33:**
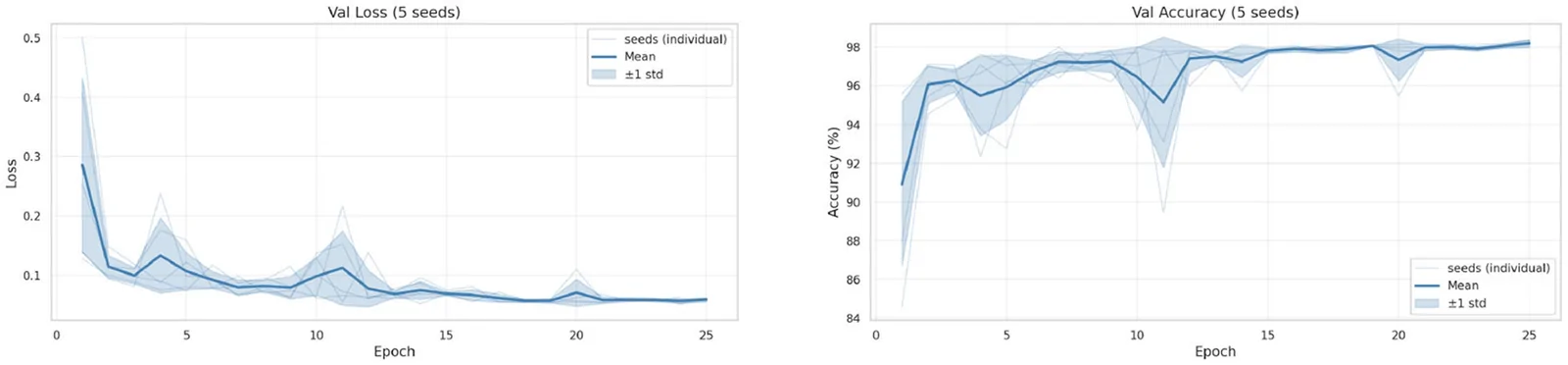
Validation losses **(Left)** and accuracies **(Right)** for the Chebyshev-CNN across 25 epochs on Data Type 1.

**Figure 34:**
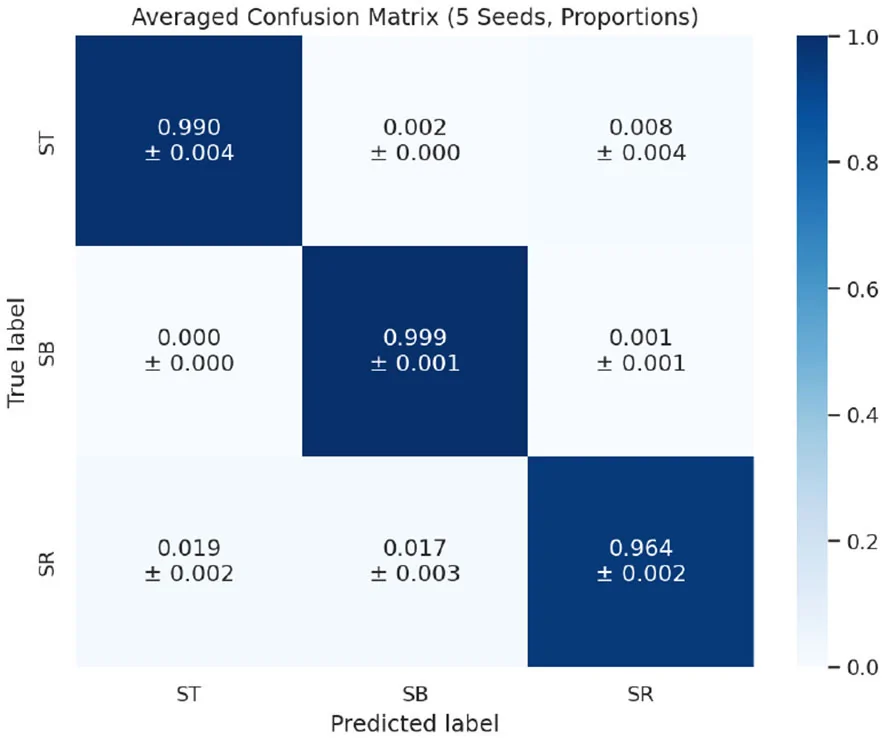
Confusion matrix for Chebyshev-CNN classification on Data Type 1.

**Table 21:** Classification report for the Chebyshev-encoded CNN on Data Type 1.

Class	Precision	Recall	F1-score	Support
ST	0.9690 ± 0.0024	0.9898 ± 0.0041	0.9793 ± 0.0013	449
SB	0.9824 ± 0.0026	0.9987 ± 0.0015	0.9905 ± 0.0012	748
SR	0.9937 ± 0.0019	0.9644 ± 0.0023	0.9788 ± 0.0015	748
Accuracy			0.9834 ± 0.0013	1,945
Macro Avg	0.9817 ± 0.0010	0.9843 ± 0.0013	0.9829 ± 0.0012	1,945
Weighted Avg				1,945

#### 3.8.2 Model performance on Type 2

For Data Type 2, which includes increased signal variability due to comorbidities, the Chebyshev-encoded CNN exhibited stable and effective convergence (Figure 35). Evaluated across five random seeds, the model achieved an average test accuracy of 97.53%±0.12%. The confusion matrix in Figure 36 confirms strong class-wise performance, with high recall values of *98.53%* ± *0.24%* for ST, *98.45%* ± *0.32%* for SB, and *95.61%* ± *0.29%* for SR, resulting in an overall test accuracy of 97.53% ± 0.12%. The detailed classification metrics are summarized in Table 22. The model achieved macro- and weighted-average precision, recall, and F1-scores of approximately **0.975**, indicating balanced performance across all three classes. The SB class attained the highest F1-score (*0.9837* ± *0.0007*), while ST and SR achieved F1-scores of *0.9758* ± *0.0016* and *0.9664* ± *0.0015*, respectively. The ROC analysis in Supplementary Figure 16a demonstrates strong discriminative capability, with AUC values of *0.9974* ± *0.0002* for ST, *0.9989* ± *0.0001* for SB, and *0.9962* ± *0.0003* for SR. The micro- and macro-averaged AUCs were 0.9973 and 0.9975, respectively, confirming reliable class separability. Finally, the micro-averaged precision–recall curve in Supplementary Figure 16b yielded a perfect average precision score of 0.9945 ± 0.0004, highlighting the robustness of the Chebyshev-based CNN even under the increased complexity of Data Type 2.

**Figure 35:**
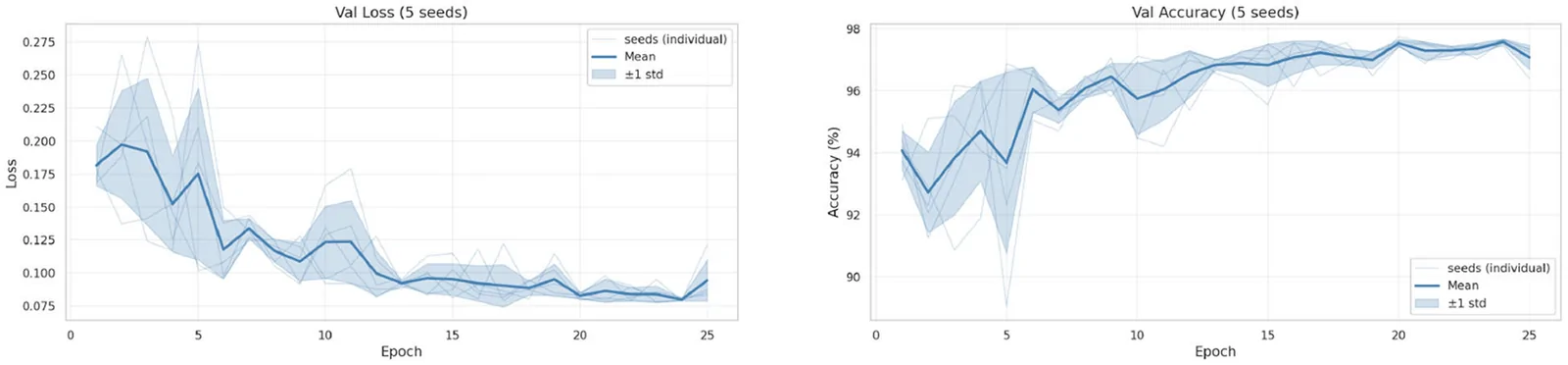
Validation losses **(Left)** and accuracies **(Right)** for the CNN using Chebyshev polynomial encoding on Data Type 2.

**Figure 36:**
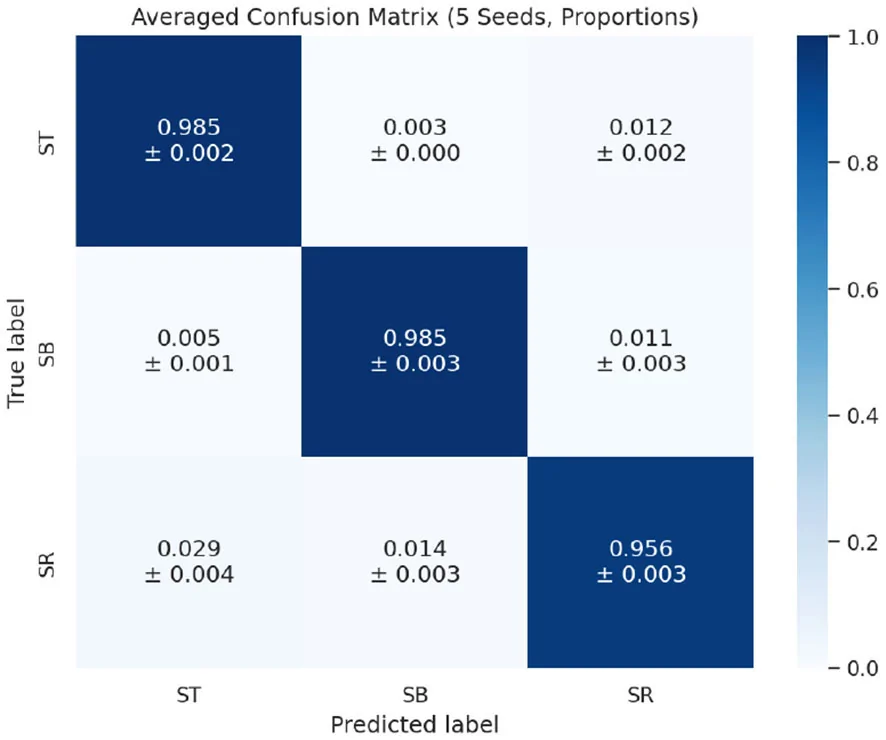
Confusion matrix for CNN classification with Chebyshev encoding on Data Type 2.

**Table 22:** Classification report for CNN with Chebyshev encoding on Data Type 2.

Class	Precision	Recall	F1-score	Support
ST	0.9665 ± 0.0038	0.9853 ± 0.0024	0.9758 ± 0.0016	748
SB	0.9830 ± 0.0026	0.9845 ± 0.0032	0.9837 ± 0.0007	750
SR	0.9768 ± 0.0022	0.9561 ± 0.0029	0.9664 ± 0.0015	748
Accuracy			0.9753 ± 0.0011	2,246
Macro Avg	0.9754 ± 0.0011	0.9753 ± 0.0011	0.9753 ± 0.0011	2,246
Weighted Avg	0.9754 ± 0.0011	0.9753 ± 0.0011	0.9753 ± 0.0011	2,246

### 3.9 Invertibility

Our encoding method is fully invertible prior to smoothing, allowing the original ECG leads to be efficiently reconstructed from the superposed image (Figure 37). To quantify this invertibility (Figure 38), we report reconstruction metrics (Table 23) such as mean squared error(MSE), mean absolute error(MAE), and relative ℓ_2_ error. It is important to note, however, that once the image resolution is reduced through smoothing or coarse-graining, the representation is no longer strictly invertible.

**Figure 37:**
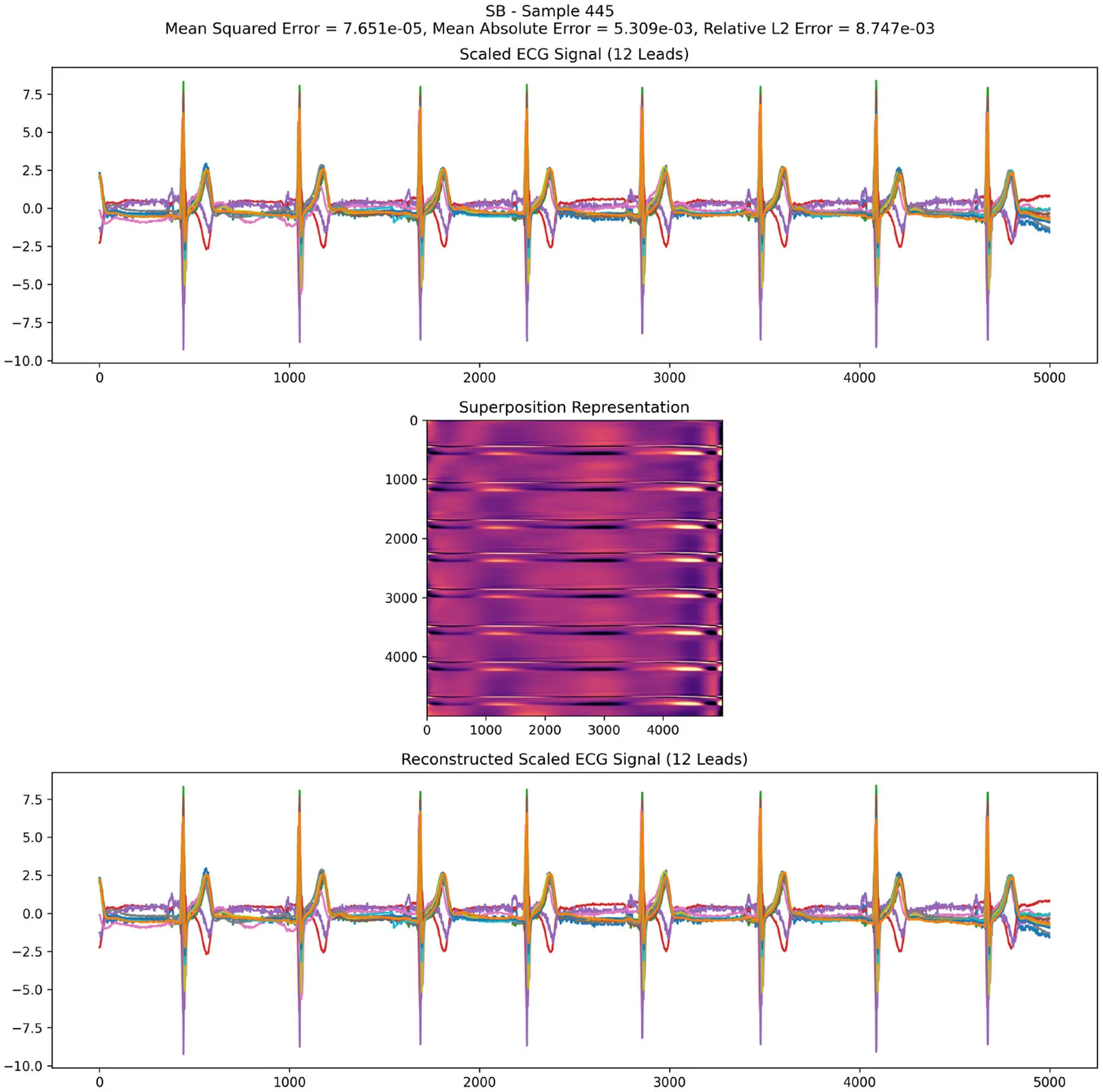
Demonstration of invertibility of the Orthogonal Polynomial Imaging (OPI) encoding prior to smoothing. **(Top)** Original scaled 12-lead ECG signal for a representative sinus bradycardia (SB) sample. **(Middle)** Two-dimensional superposition image obtained via orthogonal polynomial encoding. **(Bottom)** Reconstructed 12-lead ECG signals recovered using the analytical inverse map. The close agreement between the original and reconstructed signals, quantified by low MSE, MAE, and relative ℓ_2_ error, confirms the near-exact invertibility of the encoding before coarse-graining. The superposition image is displayed using the same pseudocolor visualization for clarity.

**Figure 38:**
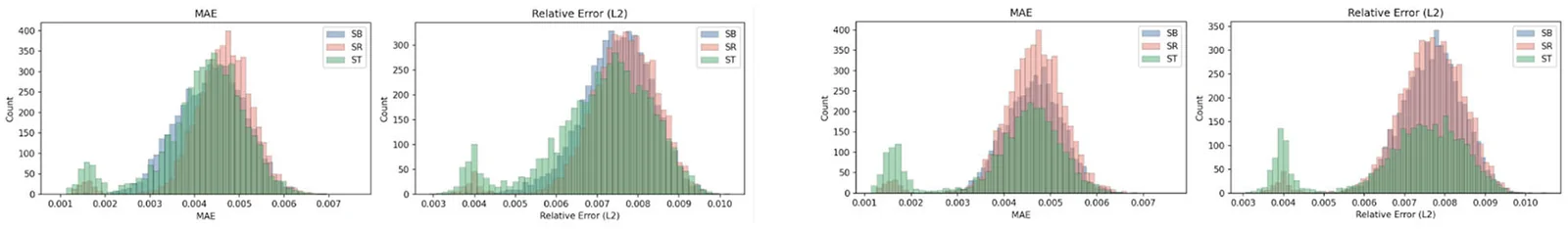
Error distributions for invertibility across the TYP1 and TYP2 datasets, evaluated using MAE and relative ℓ_2_ error.

**Table 23:** Invertibility metrics of the OPI encoding before smoothing. Reported values are averaged across the whole dataset for each rhythm class.

Class	MSE	MAE	Relative ℓ_2_ error
ST (Sinus Tachycardia)	5.0 × 10^−5^	4.09 × 10^−3^	6.99 × 10^−3^
SB (Sinus Bradycardia)	6.0 × 10^−5^	4.63 × 10^−3^	7.68 × 10^−3^
SR (Sinus Rhythm)	5.8 × 10^−5^	4.59 × 10^−3^	7.58 × 10^−3^

## 4 Discussion

### 4.1 Comparison of encoding methods

To evaluate the effect of different basis functions on model performance, we compared CNN classifiers trained on PhysioNet ECG data encoded with Legendre, and Chebyshev polynomials. Alongside accuracy, we report the Macro F1-Score (balance of precision and recall), Macro AUC-ROC (threshold-independent discrimination), and Macro Recall (clinical sensitivity). Results are presented separately for Data Type 1 (clean, single-diagnosis) and Data Type 2 (comorbidity cases). The full benchmarking results across all architectures on Data Type 1 are summarized in Table 24.

**Table 24:** Model performance comparison on Data Type 1 (Clean, Single-Diagnosis Dataset).

Method	Model	Accuracy	F1 macro	AUC	AP
Legendre CNN (100 × 100) (3.1.1)	CNN (2.2.2)	98.58% ± 0.13%	0.9853 ± 0.0015	0.9992 ± 0.0000	0.9989 ± 0.0001
Legendre CNN (50 × 50) (3.1.2)	CNN (2.2.2)	97.33% ± 0.18%	0.9718 ± 0.0016	0.9972 ± 0.0002	0.9962 ± 0.0004
Chebyshev CNN (100 × 100) (3.8.1)	CNN (2.2.2)	98.34% ± 0.13%	0.9829 ± 0.0012	0.9991 ± 0.0001	0.9986 ± 0.0002
2D ResNet (Pretrained) (3.3.2)	ResNet (2.2.4)	98.09% ± 0.39%	0.9805 ± 0.0039	0.9986 ± 0.0004	0.9980 ± 0.0006
2D ResNet (Non-Pretrained) (3.3.3)	ResNet (2.2.4)	98.23% ± 0.20%	0.9819 ± 0.0021	0.9988 ± 0.0003	0.9983 ± 0.0003
1D Transformer (3.4.2)	Transformer (2.2.3)	93.00% ± 0.75%	0.9310 ± 0.0072	0.9877 ± 0.0014	0.9793 ± 0.0026
12-Lead 1D-ResNet (3.6.1)	1D-ResNet (2.2.5)	98.40% ± 0.28%	0.9833 ± 0.0026	0.9989 ± 0.0001	0.9985 ± 0.0002
1-Lead 1D-ResNet (3.7.1)	1D-ResNet (2.2.5)	98.03% ± 0.24%	0.9799 ± 0.0023	0.9981 ± 0.0008	0.9972 ± 0.0011
Single-Lead FFNN (3.5.1)	FFNN (2.2.1)	82.30% ± 0.78%	0.8058 ± 0.0061	0.9347 ± 0.0044	0.9011 ± 0.0068

Both encoding methods achieved excellent and highly comparable performance on the clean dataset. The Legendre polynomial encoding yielded the highest peak accuracy at 98.58% ± 0.13%, with a slightly lower performance by Chebyshev at 98.34% ± 0.13%. The other metrics remained uniformly high, confirming that both polynomial families produce highly effective image representations from the clean signals. To evaluate whether the differences among top-performing models on Data Type 1 are statistically distinguishable, we conducted seed-level paired *t*-tests across all five evaluation seeds. The Legendre CNN (98.58%±0.13%) is statistically indistinguishable from the 12-lead 1D-ResNet (98.40%±0.28%, paired *t*-test *p* = 0.191), confirming that the OPI 2D image representation achieves performance parity with a raw temporal ResNet. However, the Legendre CNN achieves a statistically significant improvement over Chebyshev CNN (98.34%±0.13%, *p* = 0.0008) and non-pretrained 2D ResNet (98.23%±0.20%, *p* = 0.0026). Furthermore, the 12-lead 1D-ResNet and Chebyshev CNN remain statistically indistinguishable (*p* = 0.669). The corresponding performance comparison across all models on Data Type 2 is reported in Table 25.

**Table 25:** Model performance comparison on Data Type 2 (Comorbidities Dataset).

Method	Model	Accuracy	F1 macro	AUC	AP
Legendre CNN (100 × 100) (3.2.1)	CNN (2.2.2)	97.19% ± 0.29%	0.9718 ± 0.0029	0.9970 ± 0.0004	0.9933 ± 0.0008
Legendre CNN (50 × 50) (3.2.2)	CNN (2.2.2)	96.39% ± 0.23%	0.9639 ± 0.0023	0.9950 ± 0.0003	0.9908 ± 0.0008
Chebyshev CNN (100 × 100) (3.8.2)	CNN (2.2.2)	97.53% ± 0.12%	0.9753 ± 0.0012	0.9975 ± 0.0001	0.9945 ± 0.0004
2D ResNet (Pretrained) (3.3.4)	ResNet (2.2.4)	97.50% ± 0.16%	0.9749 ± 0.0016	0.9975 ± 0.0004	0.9956 ± 0.0009
2D ResNet (Non-Pretrained) (3.3.5)	ResNet (2.2.4)	97.39% ± 0.26%	0.9739 ± 0.0025	0.9970 ± 0.0002	0.9946 ± 0.0009
1D Transformer (3.4.3)	Transformer (2.2.3)	91.33% ± 1.07%	0.9136 ± 0.0104	0.9836 ± 0.0022	0.9689 ± 0.0053
12-Lead 1D-ResNet (3.6.2)	1D-ResNet (2.2.5)	97.78% ± 0.20%	0.9778 ± 0.0020	0.9983 ± 0.0002	0.9969 ± 0.0004
1-Lead 1D-ResNet (3.7.2)	1D-ResNet (2.2.5)	97.28% ± 0.12%	0.9727 ± 0.0013	0.9969 ± 0.0006	0.9948 ± 0.0007
Single-Lead FFNN (3.5.2)	FFNN (2.2.1)	77.52% ± 0.39%	0.7760 ± 0.0033	0.9112 ± 0.0033	0.8545 ± 0.0061

For the more complex dataset containing comorbidities, both encodings again performed comparably well, though the ranking between them reversed. The Legendre and Chebyshev encodings delivered similar peak accuracies of 97.19% ± 0.29% and 97.53% ± 0.12%, respectively, with the Chebyshev polynomial encoding performing slightly better.

### 4.2 Sensitivity to lead-to-polynomial assignment

To evaluate whether the performance of the Orthogonal Polynomial Imaging (OPI) framework depends on the specific mapping between ECG leads and polynomial basis functions, we conducted an empirical permutation experiment. We evaluated five distinct lead-to-polynomial assignments (Permutation 0 representing the default order *P*_0_ … *P*_11_, alongside four randomly generated lead permutations, Permutations 1–4) using the Legendre CNN (100 × 100) architecture across five random evaluation seeds for both Data Type 1 and Data Type 2. As shown in Table 26, performance remains highly consistent across all five lead assignments on both clean (Type 1) and comorbidity (Type 2) datasets. On Data Type 1, mean classification accuracy ranges within a narrow band of 98.19%−98.58%, while on Data Type 2, accuracy spans 96.92%−97.35%. Paired *t*-tests between each permuted assignment and the default order reveal no statistically significant differences (*p* > 0.05). These results empirically confirm that the spatial inductive bias of the 2D CNN effectively extracts cross-lead features regardless of the specific ordering of spatial polynomial basis functions, establishing that the OPI encoding is robust and shows no statistically significant dependence on lead ordering.

**Table 26:** Classification performance across five distinct lead-to-polynomial assignments (Permutation 0 = Default, Permutations 1–4 = Random Shuffles) on Data Type 1 and Data Type 2 across 5 random evaluation seeds.

Permutation	Accuracy (%)	F1 macro	F1 micro	AUC macro	AP Micro
Data Type 1 (Clean dataset)					
Permutation 0 (Default)	98.58 ± 0.13	0.9853 ± 0.0015	0.9858 ± 0.0013	0.9992 ± 0.0000	0.9989 ± 0.0001
Permutation 1	98.30 ± 0.10	0.9822 ± 0.0010	0.9830 ± 0.0010	0.9988 ± 0.0002	0.9984 ± 0.0001
Permutation 2	98.19 ± 0.30	0.9809 ± 0.0031	0.9819 ± 0.0030	0.9986 ± 0.0005	0.9981 ± 0.0005
Permutation 3	98.29 ± 0.27	0.9822 ± 0.0027	0.9829 ± 0.0027	0.9988 ± 0.0007	0.9983 ± 0.0008
Permutation 4	98.55 ± 0.04	0.9847 ± 0.0005	0.9855 ± 0.0004	0.9991 ± 0.0001	0.9987 ± 0.0001
Data Type 2 (Comorbidities dataset)					
Permutation 0 (Default)	97.19 ± 0.29	0.9718 ± 0.0029	0.9719 ± 0.0029	0.9970 ± 0.0004	0.9933 ± 0.0008
Permutation 1	97.24 ± 0.18	0.9724 ± 0.0018	0.9724 ± 0.0018	0.9973 ± 0.0002	0.9939 ± 0.0004
Permutation 2	97.34 ± 0.21	0.9733 ± 0.0021	0.9734 ± 0.0021	0.9972 ± 0.0003	0.9937 ± 0.0010
Permutation 3	96.92 ± 0.19	0.9692 ± 0.0019	0.9692 ± 0.0019	0.9968 ± 0.0004	0.9936 ± 0.0007
Permutation 4	97.35 ± 0.27	0.9734 ± 0.0027	0.9735 ± 0.0027	0.9970 ± 0.0003	0.9939 ± 0.0006

### 4.3 Comparison with past work

Recent work has increasingly focused on leveraging *12-lead ECGs with machine learning* to improve automated diagnosis. Gedon et al. (2021) explored a self-supervised pretraining strategy in which a U-ResNet model reconstructs masked subsequences across all 12 leads, thereby learning generalizable representations from the large unlabeled CODE dataset. When fine-tuned on labeled datasets such as PTB-XL and CPSC, the pretrained model showed modest but consistent improvements (e.g., AUC 0.953 → 0.954 on CPSC) and enhanced training stability, highlighting the potential of self-supervision for ECG analysis in low-label scenarios.

Building on this direction, Hu et al. (2023) proposed *ECG-MAE*, a generative self-supervised framework that masks up to 75% of spatiotemporal patches—across both time and leads—and reconstructs them to learn robust representations. They introduced a hybrid CNN–Transformer encoder (MLE-ViT) specifically designed for multi-lead ECGs, and pretrained it on large corpora (CinC2020, Chapman) before fine-tuning on PTB-XL. This approach achieved state-of-the-art performance, with macro-AUC improving from 0.9325 (supervised) to *0.9474* after pretraining, demonstrating both stronger generalization and better label efficiency compared to purely supervised and contrastive self-supervised methods.

Ribeiro et al. (2020) presented one of the earliest large-scale applications of deep learning to 12-lead ECGs, proposing an end-to-end ResNet-based model for automatic diagnosis of six common abnormalities. Trained on more than 2.3 million ECG exams from Brazil's Telehealth Network (CODE study), the network processed raw ECG signals sampled at 400 Hz and standardized to 4096 samples per lead. The model achieved high diagnostic accuracy, with F1 scores above 87% and specificity exceeding 99% across all classes. In head-to-head comparisons, the DNN matched or outperformed cardiology residents and medical students, with error analysis showing that network errors often arose from borderline threshold cases, whereas humans made more conceptual or attentional mistakes. This work provided compelling evidence for the clinical viability of deep learning in ECG interpretation, particularly in large-scale or resource-limited settings.

Taking a different perspective, Ozaltin and Yeniay (2023) proposed a framework that *transforms 1D ECG signals into 2D images (scalograms)* using continuous wavelet transform (CWT) and short-time Fourier transform (STFT). These image-based representations were classified using a custom 34-layer CNN, and further enhanced by combining the CNN as a deep feature extractor with an SVM classifier. This hybrid CNN–SVM achieved very high performance, reaching *99.21% accuracy* with CWT-based scalograms, thereby demonstrating the effectiveness of converting ECG signals into images for deep learning–based classification.

More recently, Sadik et al. (2023) proposed a geometric image representation of ECG signals by combining two leads through an outer-product construction, enabling beat-by-beat arrhythmia classification with a conventional 2D CNN. Their results further demonstrated the promise of image-based ECG representations while preserving clinically interpretable waveform characteristics. In contrast, our Orthogonal Polynomial Imaging (OPI) framework extends this line of work by encoding all 12 ECG leads simultaneously into a single structured image through orthogonal polynomial superposition, providing a mathematically principled multi-lead representation that preserves lead-specific information prior to coarse-graining.

### 4.4 Conclusion

This work introduces a framework for transforming multi-lead ECG signals into two-dimensional image representations through orthogonal polynomial superposition and coarse-graining. Inspired by techniques from high energy physics, the proposed encoding preserves the essential structure of the original signals while enabling image-based deep learning. Our extensive experiments across multiple datasets and model families establish three key findings. First, convolutional neural networks (CNNs) operating on polynomial-encoded images demonstrated that the proposed OPI representation can achieve performance comparable to strong temporal baselines, exceeding 98% accuracy on clean single-diagnosis data (Type 1) and maintaining robust performance (96–97%) in the presence of comorbidities (Type 2). In contrast, feed-forward neural networks trained on single-lead signals lagged behind, highlighting the diagnostic advantage of multi-lead integration. Comparisons with pretrained ResNet models yielded comparable classification accuracies but required substantially larger models and longer training times (Table 27). The proposed OPI representation also maintained strong performance under aggressive coarse-graining, demonstrating that compact image representations can remain highly effective for ECG classification. Similarly, models employing 1D attention mechanisms were unable to match the accuracy of the OPI-based approaches despite using larger and more complex network architectures.

**Table 27:** Computational cost and training time comparison across models and datasets (averaged over 5 runs).

Method	Dataset	Mean epoch time (s)	Mean total training time (s)
Legendre CNN (100 × 100)	Type 1	5.20 ± 0.01	123.66 ± 13.93
Legendre CNN (100 × 100)	Type 2	6.13 ± 0.00	107.77 ± 34.26
Legendre CNN (50 × 50)	Type 1	1.60 ± 0.00	33.06 ± 6.60
Legendre CNN (50 × 50)	Type 2	1.91 ± 0.04	46.51 ± 3.77
Chebyshev CNN (100 × 100)	Type 1	5.27 ± 0.01	117.43 ± 25.11
Chebyshev CNN (100 × 100)	Type 2	6.13 ± 0.00	150.59 ± 5.45
2D ResNet (Pretrained)	Type 1	50.56 ± 0.03	1051.69 ± 316.54
2D ResNet (Pretrained)	Type 2	57.94 ± 0.07	1239.63 ± 239.90
2D ResNet (Non-Pretrained)	Type 1	50.30 ± 0.03	1086.35 ± 193.37
2D ResNet (Non-Pretrained)	Type 2	59.20 ± 0.03	1136.29 ± 241.38
1D Transformer	Type 1	6.76 ± 0.02	143.27 ± 25.74
1D Transformer	Type 2	7.79 ± 0.02	160.19 ± 40.74
12-Lead 1D-ResNet	Type 1	249.32 ± 0.26	5236.79 ± 1238.13
12-Lead 1D-ResNet	Type 2	290.59 ± 0.23	6799.36 ± 753.66
1-Lead 1D-ResNet	Type 1	21.45 ± 0.02	373.24 ± 76.54
1-Lead 1D-ResNet	Type 2	24.53 ± 0.04	470.95 ± 47.66
Single-Lead FFNN	Type 1	1.15 ± 0.01	28.42 ± 0.49
Single-Lead FFNN	Type 2	1.29 ± 0.00	32.28 ± 0.16

Second, our comparative analysis of Legendre and Chebyshev encodings revealed uniformly strong results. This suggests that while the specific basis function can fine-tune performance, the broader principle of orthogonal polynomial superposition is highly robust. Third, we demonstrated that coarse-graining to lower resolutions (e.g., 50 × 50) retained excellent discriminative power, offering an efficient trade-off between accuracy and computational cost, which is crucial for real-time clinical deployment.

Relative to prior approaches, including direct raw waveform modeling with ResNets and image-based representations such as wavelet scalograms, our method offers a conceptually simple and generalizable encoding framework. However, the present study does not include a direct experimental comparison with alternative image-encoding approaches, and therefore no conclusions regarding superiority over such methods should be drawn. Unlike handcrafted feature pipelines or purely self-supervised pretraining schemes, our framework provides a physics-inspired, interpretable bridge between raw signals and deep learning architectures. Importantly, the confusion matrices and ROC analyses across experiments consistently show that sinus bradycardia is the most separable class, while sinus tachycardia and sinus rhythm exhibit higher overlap.

Looking ahead, several directions remain open. Integrating self-supervised or contrastive pretraining may improve generalization in low-label settings, while attention-based models could enhance interpretability by identifying the leads and temporal regions driving diagnostic decisions. Extending the framework to multi-label classification, rare arrhythmias, and longitudinal monitoring would broaden its clinical applicability. Beyond cardiology, the proposed encoding may also prove useful for multichannel time-series data in fields such as seismology, speech recognition, and financial forecasting. Finally, OPI is compatible with standard 12-lead ECG formats and existing clinical workflows, while its compact, partially invertible representation supports efficient inference and *post-hoc* interpretability.

While OPI preserves diagnostically relevant information and achieves performance comparable to strong temporal baselines, the present study does not isolate a representation-specific advantage arising from multi-lead encoding. While the lead-to-polynomial assignment was initially fixed, our empirical evaluations across permuted lead assignments (Table 26) confirm that classification performance exhibits no statistically significant dependence on lead ordering (*p* > 0.05). Exploring adaptive polynomial orders and dynamic basis constructions remains an important direction for future work.

In summary, we have introduced a physics-inspired framework for encoding multi-lead ECGs into compact image representations suitable for modern vision models. Our results show that orthogonal polynomial encoding, combined with convolutional architectures, provides an effective and scalable approach to ECG classification. We hope this work motivates further exploration of principled representations for multichannel biomedical signals.

## Data Availability

The original contributions presented in the study are included in the article/Supplementary material. The source code and reproduction scripts for the Orthogonal Polynomial Imaging (OPI) framework are publicly available on GitHub at: https://github.com/bloodykunu39/ECG_ML. The raw ECG recordings were obtained from the publicly available PhysioNet ECG Arrhythmia Database. Further inquiries can be directed to the corresponding author.
